# Robustness of Local Predictions in Atomistic Machine Learning Models

**DOI:** 10.1021/acs.jctc.3c00704

**Published:** 2023-11-10

**Authors:** Sanggyu Chong, Federico Grasselli, Chiheb Ben Mahmoud, Joe D. Morrow, Volker L. Deringer, Michele Ceriotti

**Affiliations:** † Laboratory of Computational Science and Modeling, Institute of Materials, École Polytechnique Fédérale de Lausanne, Lausanne 1015, Switzerland; ‡ Department of Chemistry, Inorganic Chemistry Laboratory, University of Oxford, Oxford OX1 3QR, U.K.

## Abstract

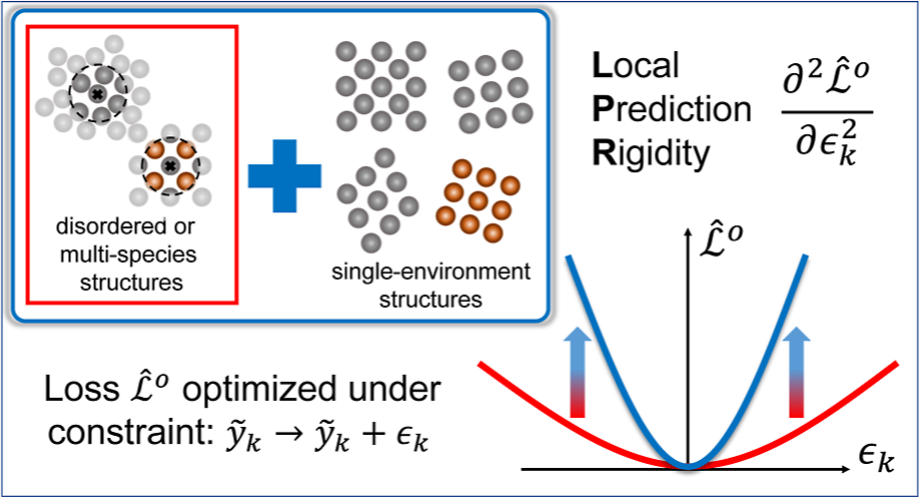

Machine learning (ML) models for molecules and materials commonly rely on a decomposition of the global target quantity into local, atom-centered contributions. This approach is convenient from a computational perspective, enabling large-scale ML-driven simulations with a linear-scaling cost and also allows for the identification and posthoc interpretation of contributions from individual chemical environments and motifs to complicated macroscopic properties. However, even though practical justifications exist for the local decomposition, only the global quantity is rigorously defined. Thus, when the atom-centered contributions are used, their sensitivity to the training strategy or the model architecture should be carefully considered. To this end, we introduce a quantitative metric, which we call the local prediction rigidity (LPR), that allows one to assess how robust the locally decomposed predictions of ML models are. We investigate the dependence of the LPR on the aspects of model training, particularly the composition of training data set, for a range of different problems from simple toy models to real chemical systems. We present strategies to systematically enhance the LPR, which can be used to improve the robustness, interpretability, and transferability of atomistic ML models.

## 1 Introduction

Extensive properties of matter, such as the total energy, arise from the collective interactions between atoms and can be rigorously defined only as *global* quantities that depend on the entire molecule or the condensed-phase structure. Nonetheless, the last decades have seen considerable efforts toward the construction of quantum-chemical methods that exploit the quantum-mechanical nearsightedness principle^1^ to perform a *local* decomposition of the global quantities.^2−7^ These methods either undertake a physically motivated local decomposition in the calculation of a global quantity^8−11^ or perform such decomposition for the purpose of analysis.^12−17^ Despite the fact that the local quantities are not physical observables, such a decomposition allows one to break down the macroscopic observable for a complex structure into contributions from much simpler components, typically individual atoms and their neighbors. Consequently, such methods have led to drastic improvements in the time and cost scaling of quantum-mechanical calculations and allowed researchers to gain an enhanced understanding of the physical and chemical nature of materials.^18−22^

The idea of decomposing a global quantity into contributions associated with local environments has also become a cornerstone of atomistic machine learning (ML).^23−27^ ML models can be trained to predict the contributions of the local environments to the global quantity of interest, which are then summed to ultimately yield the global prediction for a target system. Within the context of ML, this approach has two distinct advantages, the first of which is scalability. Local decomposition allows the models to be easily applied to systems of vastly different length scales (training on small cells and predicting for much larger ones),^23,24^ underpinning their widespread usage. This is especially the case for ML interatomic potentials,^25,28−32^ which allow accessing longer length and time scales in simulations with a linear-scaling cost.

The second advantage is that contributions from a local, machine-learned decomposition of the global quantity can offer considerable heuristic power because one can then use the ML model to describe the complex behavior of chemical systems as resolved according to the local contributions from their constituent building blocks. Having access to such “local predictions” has enabled the development of ML models for the prediction of thermal transport in electronic insulators,^33−39^ where the locally predicted energies are needed in classical-like expressions of the heat flux^40^ used in Green–Kubo theory. In the case of ML models of the electronic density of states,^41−45^ a plausible correlation could be found between different local structural motifs and how they “contribute” to the total density of states. More recently, researchers have been actively exploiting these locally predicted values to interpret the local stability of chemical environments in complex phases,^46−50^ guide structural optimization,^51^ and even use them as synthetic data for the pretraining of large neural network (NN) models.^52^

While the practical benefits of local decomposition for atomistic ML are clear, one must be mindful of how reliable, or “robust”, the resulting local predictions are. Since only the global quantity is rigorously defined, its decomposition into local contributions can take place in numerous different ways.^53^ Then, if the local predictions of an ML model are sensitive to the smallest changes in model training (e.g., subsampling of the same data set), their reliability would be compromised, along with that of any interpretation that has been made using these predictions. Also, excessive sensitivity to model training details often indicates that extrapolative predictions are unstable, which translates to poor transferability of the resulting models.^54^ It is therefore of significant interest for the atomistic ML practitioners to understand how reliable and robust the local predictions of their ML model are and what can be done to improve them.

In the present work, we propose a new metric, which we refer to as the local prediction rigidity (LPR), that quantifies the robustness of local predictions made by atomistic ML models. Through a series of case studies on different models, we uncover the existence of varying degrees of robustness in the local predictions, which primarily depend on the composition of the data set used for model training. We further demonstrate strategies by which the LPR can be systematically enhanced for the local environments of interest, which can ultimately improve the overall robustness, interpretability, and transferability of atomistic ML models.

## 2 Theory

Consider a generic ML model that predicts the global property *Y* of a structure *A* by summing the predictions for individual atom-centered contributions, *ỹ*. The task for model training is to minimize the loss function, , which quantifies the difference between the reference values *Y*_*A*_ and the global ML model predictions
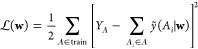
1

The set of optimized coefficients **w**^*o*^ that minimizes  is obtained by setting the derivative of  with respect to **w** equal to 0. Close to **w**^*o*^, one can approximate *ỹ* by a second-order Taylor expansion
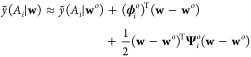
2where  is defined as
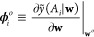
3and  as
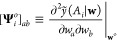
4

With this approximation, one can also expand the loss around **w**^*o*^ up to the second order
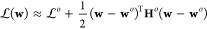
5

Here, , and
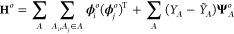
6is the Hessian of the loss evaluated at **w**^*o*^, with  and . Note that no linear term in (**w** – **w**^*o*^) appears in eq 5 because of the optimization condition.

To assess the robustness of local predictions made by an ML model, one can consider how sensitive the model is to a change ϵ_*k*_ in a local prediction associated with an arbitrary environment *k*. To do so, however, explicit control over the model prediction is needed. For this purpose, one can consider the following modified loss function, which incorporates a Lagrangian term that constrains the model prediction for a local environment *k* to be perturbed by ϵ_*k*_
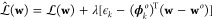
7

Minimization of the new loss leads to
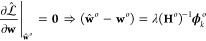
8where  is the new array of optimal weights. By enforcing the local prediction constraint , the following expressions for λ and ( – **w**^*o*^) can be obtained
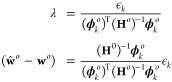
9

These expressions lead to algebraic simplifications, resulting in the following expression for the optimized constrained loss, where the dependence on ϵ_*k*_ is now explicitly enforced
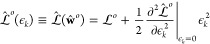
10

The term
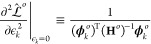
11is the second derivative of the constrained, optimized loss with respect to the change ϵ_*k*_ in the local prediction and where we used . Note that in cases where regularization of the weights is performed, the derived expressions will differ only by the inclusion of an additional regularization term in the loss and in **H**^*o*^.

Ultimately, ∂^2^/∂ϵ_*k*_^2^ describes how sensitive the model is to perturbations in a given local prediction, via the changes in **w** caused by these perturbations. A large value of ∂^2^/∂ϵ_*k*_^2^ indicates that the corresponding local prediction has been robustly made as its perturbation steeply increases the loss and severely penalizes the model. Conversely, small ∂^2^/∂ϵ_*k*_^2^ indicates that the corresponding local predictions are less robust. Since ∂^2^/∂ϵ_*k*_^2^ essentially captures how “rigid” a given local prediction is, it is hereon referred to as local prediction rigidity or LPR for short.

Having derived the LPR for a generic ML model, one can make further substitutions to obtain the expression for a specific type of model. For a linear model
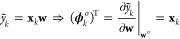
12where **x**_*k*_ is a row vector containing the features of environment *k*. The Hessian reads
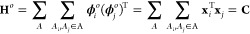
13where **C** = **X**^T^**X** is the covariance of the feature matrix **X** of the training set, whose rows  are the feature vectors of each structure. Note, also, that the second term on the right-hand side of eq 6 vanishes since the predictions are linear in the weights. Therefore, for the linear model
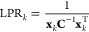
14

As already mentioned, when an L^2^ regularization with regularizer strength μ is added to the loss, it is sufficient to set .

For a sparse kernel model with L^2^ regularization, the following expressions are obtained from direct substitution
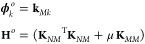
15where we adopt the notations from ref (55) in which *N* indicates the training set and *M* indicates the active set. This means that, for the sparse kernel model, the LPR of the local environment *k* is
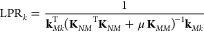
16

In both models, the LPR depends solely on the composition of the training set and not on the actual loss or target quantities. Such an exclusive dependence on the makeup of the training set hints at the crucial importance of judiciously composing the training structures to improve the level of robustness in the local predictions.

Here, one should recognize that this property is also shared, in the context of Gaussian process regression (GPR), by estimators of the uncertainty of a prediction. For instance, in the subset of regressor (SR) approximation,^56^ one can express the uncertainty as
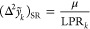
17where, again, μ is the regularizer strength. Similar relations follow for other uncertainty estimates.^1^ It is interesting to see that in all cases, the LPR-containing term exclusively captures the dependence of Δ^2^*ỹ*_*k*_ on the composition of the training set, as seen through the lens of the features, or the kernel, used by the model.

So far, we have constructed all of the main theoretical elements to quantitatively describe the robustness of a prediction for a given local environment, which in itself is not a physical observable. Here, we briefly note that in the limiting case of a structure consisting of a single type of local environment (e.g., crystalline structures in which a single Wyckoff position is occupied), the local prediction has a well-defined target of *ỹ*_*k*_ = *Y*_*A*_/*N*_*A*_ and should therefore exhibit a maximal LPR value: any change to it would result in a change in the prediction of the global quantity of the entire structure, with a direct increase in  that is consequential. On the contrary, in disordered structures or structures containing atoms of different species, the local predictions would generally be far less robust and exhibit much lower LPR values due to the degeneracy in the ways in which the global quantity can be partitioned. In the following sections, we demonstrate how the LPR becomes defined for the general case and also propose strategies that can systematically improve the LPR and the robustness of local predictions made by atomistic ML models.

## 3 Proof-of-Concept Using Toy Models

To establish and demonstrate the concepts associated with the LPR, we first constructed and examined a toy model. This model is devised to make local predictions, *ỹ* ≡ *ỹ*(*x*), depending solely on a scalar input *x* (local features), but is trained using global targets *Y* that are the sum of contributions from multiple *x*_*k*_ values, i.e., *Y* = ∑ *ỹ*_*k*_. This formulation directly corresponds to atomistic ML models, where the model predictions are made for local environments in a structure, yet regression is performed on global quantities that correspond to the entire structure. A pseudo-data set of four data points *Y*_1,···,4_ is constructed for training. The toy model for *ỹ*(*x*) is assumed to be an eighth-order polynomial in *x* and to include an L^2^ regularization term (Figure S1).

**Figure 1:**
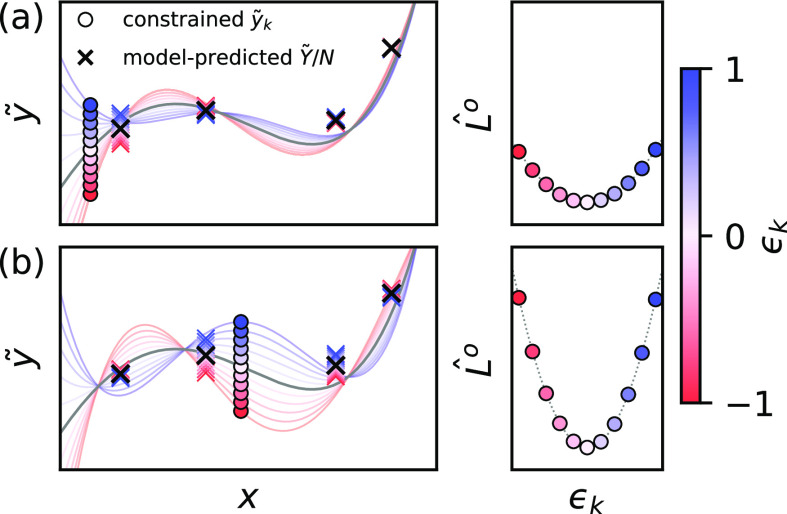
Graphical demonstration of the LPR using a numerical toy model. The left panels show, in different colors, how the model *ỹ*(*x*) changes when the prediction *ỹ*_*k*_ is changed by ϵ_*k*_. The prediction of the original, unconstrained model is colored gray, and the results for the constrained models are colored different colors that depend on ϵ_*k*_. Predictions *Ỹ* for the total target quantity are shown by crosses and normalized by the number *N* of elements of each group *X* of local features. The right panels show the resulting profile of  as dependent on ϵ_*k*_, the curvature of which corresponds to the LPR. (a) and (b) report the same analysis repeated for two distinct local features. When the ϵ_*k*_-dependent changes in *Ỹ* are small, the model readapts without affecting  much and LPR_*k*_ are low, as shown in (a). On the contrary, if substantial changes in the total predictions *Ỹ* occur,  is severely affected by ϵ_*k*_ and LPR_*k*_ is large, as presented in (b).

As a concrete demonstration of the idea behind the LPR, we train a series of toy models where, for a chosen *x*_*k*_, *ỹ*_*k*_ is incrementally constrained away from the original prediction by an amount ϵ_*k*_ (right-hand side of Figure 1). These perturbations inevitably affect the overall optimized loss  of the model. What ultimately results is a parabolic profile of  around the original prediction of *ỹ*_*k*_, the curvature of which is then quantified and interpreted as the LPR. By comparing the two cases presented in the figure, one can observe the different outcomes for different choices of *x*_*k*_: the model is far more sensitive to changes in (b) than in (a). Such a higher sensitivity captures the model tendency to retain the original local prediction and corresponds to a larger LPR. Conversely, lower sensitivity is a sign of arbitrariness in the corresponding local prediction, which is associated with a smaller LPR.

**Figure 2:**
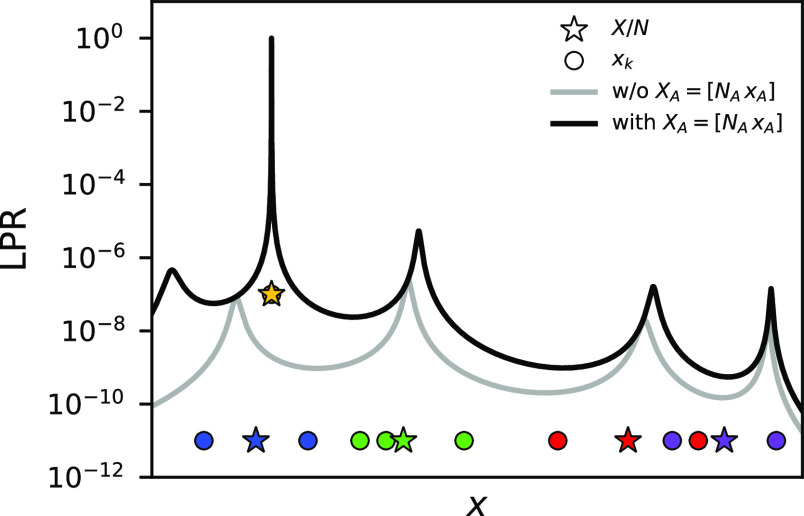
LPR profiles of a numerical toy model over the entire range of interest for local feature *x*. Values of *x*_*k*_ that appear in the training set are plotted with circles on the bottom, color-coded according to the group to which they contribute. Stars mark *X*/*N* of each global data point in the training set, which corresponds to how the global quantity would be predicted. The LPR profiles are shown for the model before (gray) and after (black) inclusion of a group of local features *X*_*A*_ = [*N*_*A*_*x*_*A*_] that consists of one local feature *x*_*A*_ replicated multiple times, shown in yellow.

Since the input value *x* of the toy model can be continuously varied, the LPR can be computed over the entire range of interest and not only for points that are part of the training set. As shown by the gray line in Figure 2, this reveals the existence of peaks in the LPR profile at which the local predictions are more robust than elsewhere. The positions of these peaks do not necessarily correspond to any particular *x*_*k*_ found in the training set nor to the average of the group *X*_*A*_ = [*x*_*A*_1__, *x*_*A*_2__,···] of local features associated with a global quantity *Y*_*A*_. Instead, as we will demonstrate later, they have a delicate dependence on the degrees of freedom associated with the decomposition of the global quantity into local contributions. It is worth noting that the regularization strength μ affects the overall range of LPR and the width of the peaks that appear (Figure S2). While regularization can therefore offer some control over the robustness of local predictions, one must keep in mind that overregularization can easily compromise the model accuracy: stable local predictions are not useful unless they lead to accurate global quantity predictions.

The analyses up to this point establish that local predictions of atomistic ML models would exhibit varying degrees of rigidity, which can be quantitatively described by using the LPR. A subsequent question arises: what is the range of possible values for the LPR? Here, we note that the lower limit of LPR can be deduced from the expected behavior of a linear model in the data-poor, overparametrized regime in the absence of regularization. In such a model,  would always be 0 for any value of ϵ_*k*_, for any *x*_*k*_ in the training set.^2^ This is because the overparameterized model would be capable of counteracting the perturbative changes in other local predictions and always retain the correct predictions for the set of global quantities. As such, LPR_*k*_ would also be 0, signifying complete arbitrariness in these local predictions.

To approach the opposite case where the LPR would instead be extremely high, we start by introducing a special class of *X*_*A*_ made of a single input *x*_*A*_ replicated *N*_*A*_ times, i.e., *x*_*A*_1__ = *x*_*A*_2__ = ... = *x*_*A*_. For such *X*_*A*_, the local prediction *ỹ*_*k*_ is directly linked to the global quantity since it must target *Y*_*A*_/*N*_*A*_. In the context of atomistic ML, *X*_*A*_ corresponds to what we later refer to as a “single-environment” structure, where all of the local environments appearing in the structure are described by the same set of features. For such cases, the change in  with a perturbation in the local prediction *ỹ*(*x*_*A*_) will be dramatic since it directly affects the prediction of the global quantity *Y*_*A*_. In fact, as shown by the black line in Figure 2, the addition to the training set of (*X*_*A*_, *Y*_*A*_) with *X*_*A*_ = [*N*_*A*_*x*_*A*_] creates a large peak in the LPR profile, which sits on top of *x*_*A*_. We remark that LPR ≈ 1 observed at the peak is not a “hard” limit as there could easily be cases where inclusion of multiple *X* groups with similar *x*_*k*_ values or strong regularization of the model leads to LPR values that surpass 1.

**Figure 3:**
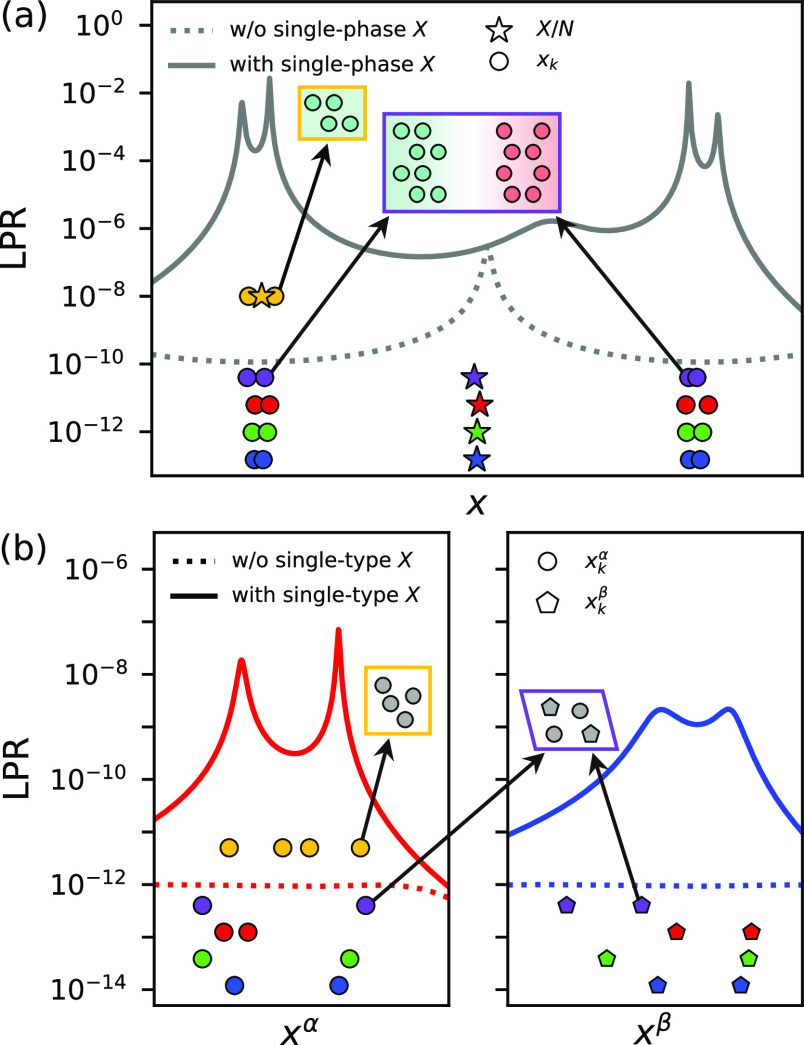
Effect of heterogeneity in the training data on the LPR, demonstrated using the toy model. (a) LPR profile of a model trained on a data set containing local feature groups *X* with a fixed composition between two phases (dotted line), which hints at the degeneracy in the local predictions for the two phases. Inclusion of a single-phase *X* (yellow) lifts the degeneracy and enhances the LPR for both phases (solid line). (b) LPR profile of a composite model trained on a data set of groups *X* containing two distinct local feature types, α and β. A data set with a fixed α/β compositional ratio results in very low LPR for both α and β (dotted line). With the addition of *X* only composed of α (yellow), the degeneracy becomes resolved, and the LPR is enhanced for both (solid line).

We now discuss two examples that illustrate the behavior of the LPR in more complicated scenarios. In the first example, we assume the existence of two distinct “phases” in the training set. This is realized by imposing a separation between two groups of local feature values, each associated with small fluctuations around one distinct value. For each *X* in the training set, the same number of local features is sampled from the two phases. A new model is then trained, and its LPR profile is computed. The profile reveals a single peak between the two phases, which is much larger than the LPR of the actual phases (Figure 3a). Subsequently, another *X* exclusively composed of local features belonging to a single phase is added to the data set. The LPR profile of the retrained model shows *two* main peaks corresponding to the two phases, as well as an overall increase in LPR.

These differences in the LPR profile are explained by how the degrees of freedom in the target quantity decomposition change. Initially, partitioning the global quantity into contributions from the two phases is completely arbitrary. That is, the local prediction for either of the two phases can be freely made as the prediction for the remaining phase can be adjusted to accurately recover the global quantity. The addition of a single-phase *X* to the data set, however, fixes the local prediction for the corresponding phase and, indirectly, also constrains the prediction for the remaining phase. In other words, the degeneracy in the partitioning of the global quantity into contributions from the two phases gets lifted. A similar mechanism is also at play in a second example in which we consider multicomponent systems, represented using a toy model with two distinct types of local features, each associated with a separate prediction function. Results in Figure 3b show that the effects of the previous example persist here as well, even though the predictions are made for *x*_*k*_ values that are completely disconnected in the feature space.

Note that in both examples there also exist further splittings of peaks in the LPR profile beyond what has been explained in terms of the phases or types. This suggests that similar effects must be taking place within each phase or type, where the remaining degrees of freedom in decomposing the global quantity are further resolved. All in all, one can expect the LPR of real atomistic ML models to be determined on similar grounds, although the way in which multiple degrees of freedom are combined together and then resolved for structures of diverse atomic compositions would easily become quite complex.

## 4 Case Studies on Demonstrative Chemical Data Sets

Having clarified the construction and the interpretation of the LPR using a toy model, we now illustrate how it can be used for actual atomistic ML models trained on chemical data sets. For this purpose, we consider three systems: amorphous silicon (a-Si), amorphous carbon (a-C), and gallium arsenide (GaAs). In all cases, we train sparse kernel models using the total energies of the structures as the target. The predictions are made by summing the contributions from all atomic environments in a given structure. The data sets are judiciously constructed to elucidate various trends that underlie the behavior of the LPR. The atomic environments are described using the smooth overlap of atomic positions (SOAP) descriptor and kernel.^57^ For demonstrative purposes, we choose hyperparameters that enhance the variation of the LPR seen in the different test cases while retaining sufficient model accuracy. As we shall see in Section 5, similar trends are also observed when hyperparameters are used that are optimized only for the model performance. Full details of the data set construction and ML model training are provided in the Supporting Information.

**Figure 4:**
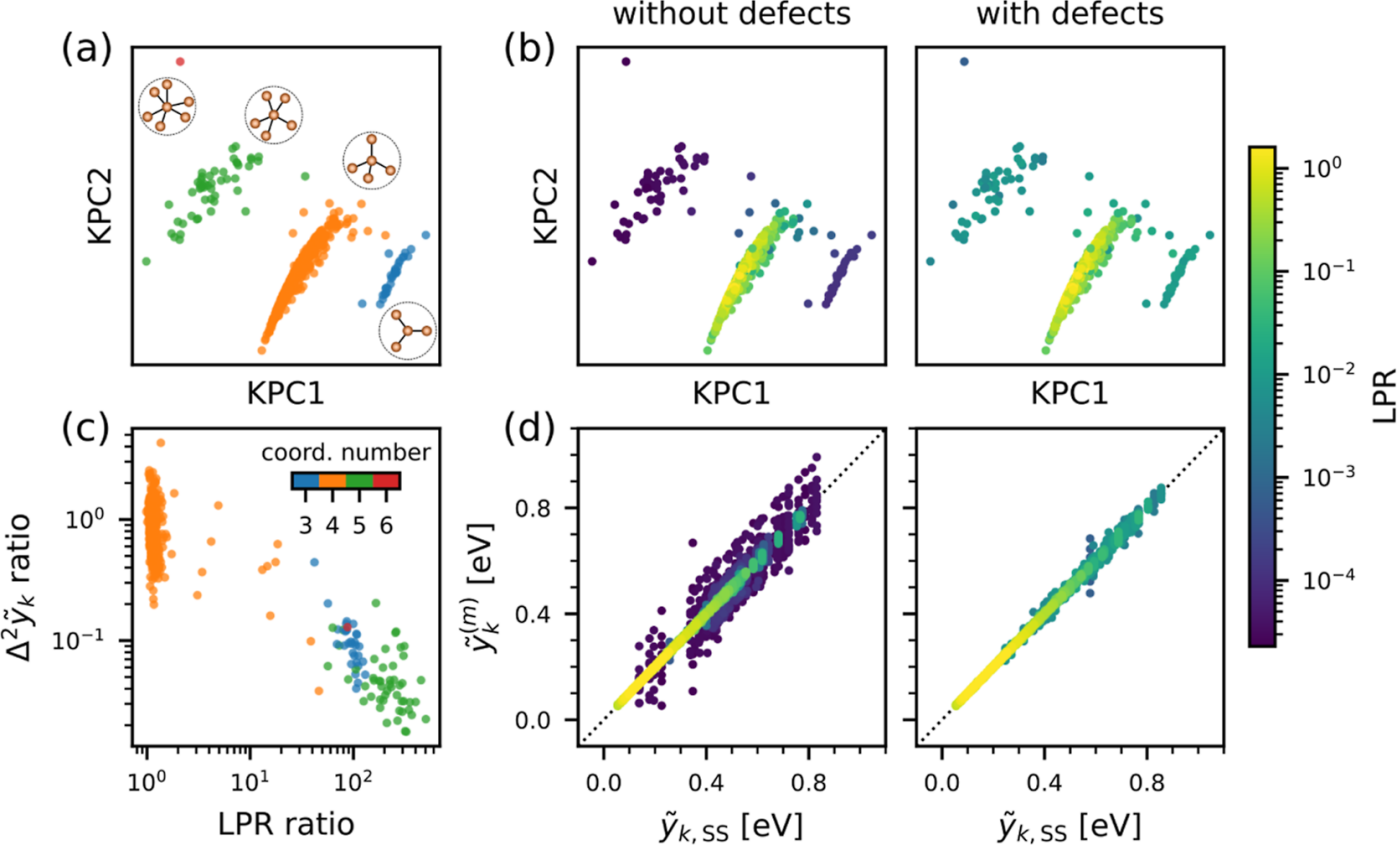
LPR and local energy predictions of models trained on the amorphous silicon (a-Si) data set before and after the inclusion of structures containing under-/overcoordinated defect environments in the training set. (a) Kernel principal component analysis (KPCA) map with the points color-coded by coordination numbers of the atomic environments. For each cluster of points, a corresponding schematic environment is shown as insets. (b) KPCA map color-coded by the LPR value from each model. (c) Ratio of the variance of the committee-predicted local energies (Δ^2^*ỹ*_*k*_) vs ratio of the LPR, before and after inclusion of the defect-containing structures in the training set. (d) Parity plots of the local energies predicted by a committee of 10 models vs the committee average prediction, where the points are color-coded by the corresponding LPR values. Energy values are reported with respect to the atomic energy of crystalline silicon.

In elemental silicon under ambient conditions, each atom normally bonds with four of its neighbors to form a tetrahedral coordination environment. While most environments in the a-Si data set are close to this ideal geometry, some are “defective”, being either under-coordinated or overcoordinated (as detected by a bond cutoff distance of 2.7 Å^3^). We study the effect of including defect-containing structures in the training set on the resulting LPR of the model. To analyze results, kernel principal component analysis (KPCA) is performed to plot the local environments in a low-dimensional representation of the feature space and then color-coded by the LPR to study the trends. Figure 4 shows that the LPR of under-/overcoordinated environments in the test set is comparatively low for an ML model trained on 500 defect-free, 64-atom structures. When 10% of the training set is replaced by the defect-containing structures, the LPR of the defect environments is enhanced by several orders of magnitude. The variance of local energy predictions across a committee of models (herein referred to as Δ^2^*ỹ*_*k*_ without any subscripts) significantly decreases for the defective environments, in line with the link between the LPR and GPR uncertainty. This is further corroborated by the change in  from eq 17, which is reduced by up to 112 meV (compared to 3–6 meV root-mean-square error (RMSE) per atom for a test set of defect-containing structures).

**Figure 5:**
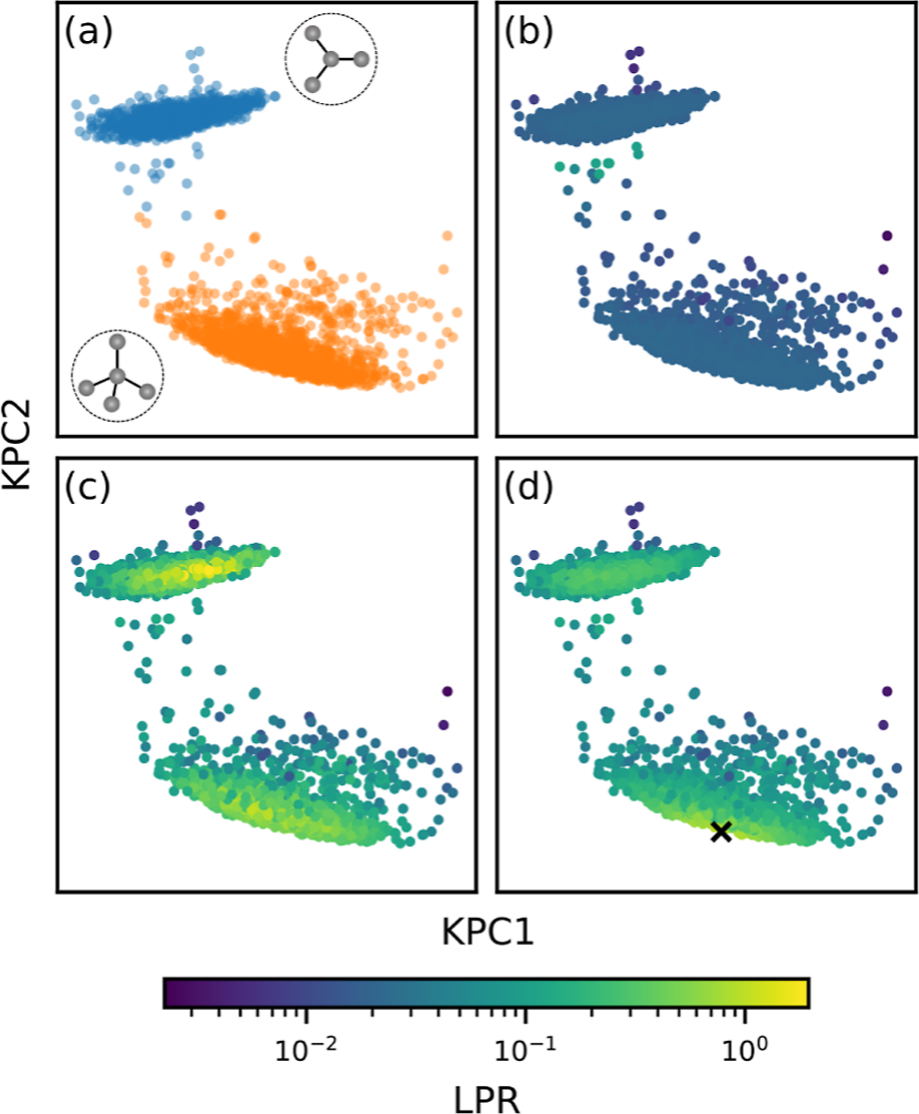
KPCA maps for an ensemble of amorphous carbon environments colored by the hybridization of the atoms, shown in (a) and then by the LPR of the models trained on differently composed training sets. The top and bottom clusters of points correspond to the sp^2^ and sp^3^ environments, respectively, and the corresponding schematic environments are shown as insets. (b) Results obtained when the model is trained on an initial data set exclusively composed of structures that retain a 1:1 ratio between sp^2^ and sp^3^ carbons. (c) 10% of the initial data set is replaced with structures exhibiting a different sp^2^ to sp^3^ ratio. (d) Single structure in the initial data set is replaced with the crystalline diamond structure, for which the location in the KPCA map is marked with a cross.

The a-C data set is composed of 500 structures containing 64 atoms that are a mixture of “sp^2^” and “sp^3^” carbons (defined by counting bonded neighbors up to a cutoff distance of 1.82 Å^4^). This effectively introduces a degree of freedom in the decomposition of the total energy into the contributions from the two distinct types of carbon environments. In fact, when the model is trained on a data set exclusively composed of structures with a 1:1 ratio between sp^2^ and sp^3^ carbons, the energy partitioning between the two carbon types is performed rather arbitrarily, as evident from the LPR (Figure 5b). Drawing on what was previously observed for the toy model on an artificial two-phase system (Figure 3a), we introduce structures that exhibit a different ratio between the two carbon types into the training set to lift the apparent degeneracy. Indeed, Figure 5c shows that when 10% of the training set is replaced by structures with a different ratio between sp^2^ and sp^3^ carbons, the LPR increases for both. The increased robustness in local energy predictions is confirmed by a notable decrease in Δ^2^*ỹ*_*k*_ (Figure S3).

Another effect that can be demonstrated with the a-C data set is the enhancement of LPR from the inclusion of high symmetry, “single-environment” structures. Both sp^2^ and sp^3^ carbons have crystalline analogues, graphite and diamond, where the local energy target is unequivocally defined as all of the atoms in the structure are described with the same set of local features due to symmetry. In Figure 5d, it is shown that the LPR improves significantly when a single crystalline diamond structure is included in the training set, especially for the sp^3^ environments that are close to diamond on the kernel PCA map. Inclusion of the diamond structure is also capable of resolving the energy decomposition degeneracy between the sp^2^ and sp^3^ carbon atoms, and hence improvement in the LPR is observed for the sp^2^ environments as well. Once again, this can be equivalently seen as the decrease of Δ^2^*ỹ*_*k*_ for both sp^2^ and sp^3^ environments (Figure S4). These results emphasize the importance of recognizing and resolving degeneracies associated with distinct phases or atomic types in a data set, which could be as simple as including a small number of single-environment structures associated with each phase/type.

**Figure 6:**
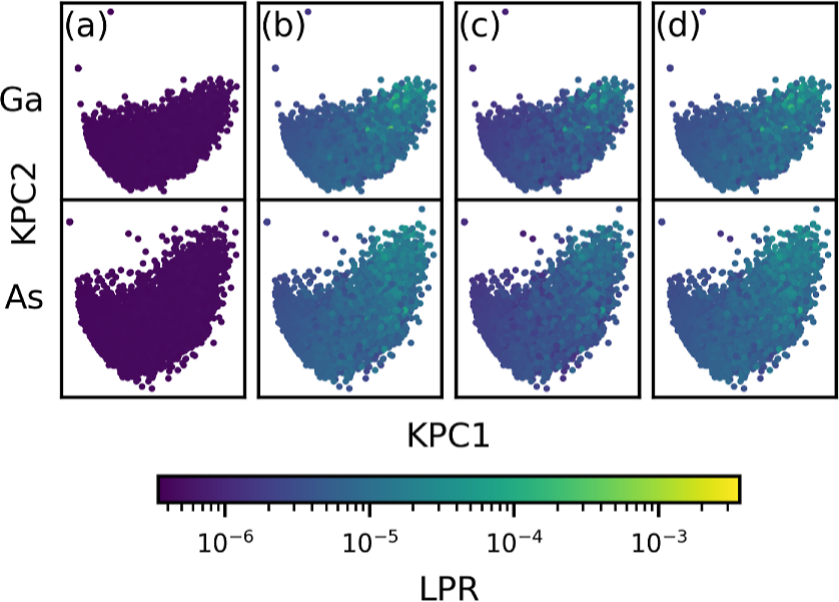
KPCA maps for a GaAs data set. Separate maps are shown for Ga (top row) and As (bottom row) atomic environments, and the points are color-coded by the corresponding LPR values. Results are shown for a series of models trained on data sets with different compositions: (a) exclusively composed of structures with a Ga/As ratio of 1:1; (b) with 10% of the data set replaced with structures exhibiting a different Ga/As ratio; and (c,d) with 10% of the data set replaced with pure Ga or pure As structures, respectively.

Finally, we explore effects in the LPR associated with the presence of multiple atomic species in the structures using a GaAs data set, a physical analogue of the toy model presented in Figure 3b. For a model trained exclusively on 400 structures of varying numbers of atoms (176 to 384) with 1:1 stoichiometric composition (Figure 6a), the LPR remains consistently low for both Ga and As and does not even show significant variations in the values within. This signifies close-to-complete arbitrariness in the energy decomposition between the two species. Note that this would have serious implications in terms of transferability: if this model was used to extrapolate on pure Ga or As structures or even on structures with a Ga or As vacancy, the predictions are likely to be nonsensical. Figure 6b–d shows the results when 10% of the training set is replaced by structures with a different Ga/As ratio, pure Ga structures, or pure As structures, respectively. In all cases, the degeneracy in the local energy decomposition is resolved, the LPR of both Ga and As is notably enhanced, and Δ^2^*ỹ*_*k*_ becomes significantly smaller (Figures S5–S7).

These case studies demonstrate that similar to what was previously observed for the toy model, robustness in the local predictions can drastically vary even for atomistic ML models trained on real chemical systems, and the degree of robustness quantified by the LPR depends on the composition of the training set. To improve the LPR and hence the robustness of the local predictions, one must first ensure a sufficient representation of all local environments of interest in the training set structures. In the case of chemical systems with distinct phases/local environments or species, the training set should be carefully composed so that the degeneracy in the energy decomposition could be resolved as much as possible. We note in closing that these effects are not specific to the sparse kernel model, as similar trends are consistently observed when the analyses are repeated for linear ridge regression models (Supporting Information).

## 5 Realistic Applications

The demonstrative case studies of Section 6 elucidate the existence of varying degrees of robustness in the local predictions made by atomistic ML models, as quantified by the LPR, and how it depends on the composition of the training set. In this section, we further expand upon our findings to devise strategies to systematically enhance the LPR and the robustness of local predictions. In the general case, the degeneracy in the local decomposition is expected to be far more complex than those seen in the previous case studies. One failsafe strategy to guarantee high LPR would be to judiciously compose the training set, from scratch, in a manner that resolves the degeneracy for as many local environments of interest as possible. In most cases, however, such an approach would be hindered by data availability and the computational cost associated with generating the necessary new data.

Here, we instead propose the generation and inclusion of single-environment structures into the training set as a simple yet effective strategy in which the LPR can be systemically enhanced. As previously discussed, single-environment structures are those composed of one local environment replicated multiple times, such as the case of single-species crystalline structures with a single Wyckoff position, which leads to an unequivocal definition of the local prediction target. This results in a maximal LPR value for the corresponding local environment and increased LPR for sufficiently similar environments around it (Figures 2 and 5d). Then, by introducing single-environment structures that closely resemble the local environments of interest to the training set, one can improve the robustness of the model predictions, as evidenced by an enhancement in the LPR. One should also note that due to their high symmetry (i.e., small and simple unit cells), generating such structures and obtaining their reference properties is considerably cheaper than constructing the rest of the data set.

To demonstrate this strategy, we present a realistic case study where the inclusion of single-environment structures in the model training enhances the LPR for the local environments of interest in the target system. For this, we direct our attention to the studies of a-C films conducted by Caro et al.^58,60^ Benefiting from the scalability of atomistic ML models, the authors carried out large-scale simulations to uncover the growth mechanism of a-C films when they are grown by the deposition of highly energetic ions onto a substrate. They also computed the GPR-based error estimates to ensure that the uncertainty in the model predictions remains reasonably low throughout their simulations. Here, we further expand on this by showing that it is possible to systematically enhance the LPR for particular local environments of interest and reduce the uncertainty in the model predictions.

The a-C films from ref (58) significantly vary in their mass densities, depending on the energies of incident atoms for deposition. The films hence exhibit different similarities in their local environments to graphite (lower density) or diamond (higher density), which are both crystalline, single-environment structures. As such, we train and analyze carbon ML models before and after the inclusion of single-environment structures obtained as high-symmetry distortions of diamond or graphite. First, we train a SOAP-based sparse kernel model with the identical set of hyperparameters used in the reference study,^30^ on 1000 randomly chosen a-C structures from the authors’ published data set. The model is subsequently retrained under the same conditions, but with 10 structures in the training set replaced with diamond and/or graphite and derivative structures. The derivative single-environment structures are generated by distorting the unit cell vectors while occupying the original, single Wyckoff position (Figure S16). This procedure ensures that while the local environment changes, all atoms in the unit cell are still described equivalently. Full details of the model training and derivative single-environment structure generation are provided in the Supporting Information.

**Figure 7:**
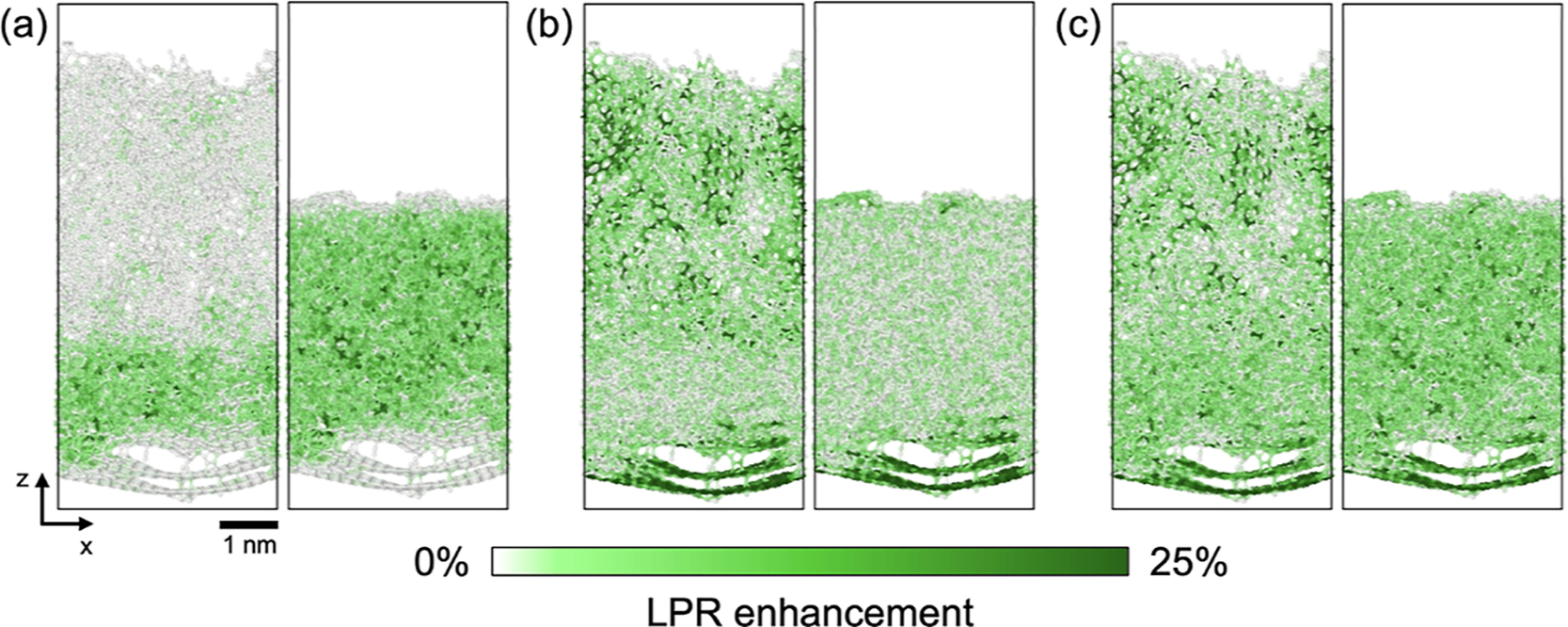
Enhancement in the LPR for low-density (left) and high-density (right) carbon films taken from ref (58) with the inclusion of single-environment structures in the training set. Results are shown for SOAP-based sparse kernel models of elemental carbon, as described in the text. In all cases, the enhancement is computed with respect to a baseline model trained on 1000 amorphous carbon structures. (a) LPR enhancement when 10 training set structures are replaced with diamond-like single-environment structures, which constitutes only 1% of the training set. The enhancement is mostly observed for the local environments of the high-density film. (b) LPR enhancement when 10 structures are replaced with graphite-like single-environment structures. The enhancement takes place for the environments found in the low-density film. (c) LPR enhancement when five diamond-like and five graphite-like structures are incorporated into the training set. The enhancement is consistently observed for the local environments of both films. Structures were visualized using OVITO.^59^ Axis labels and scale bar shown in the bottom left corner correspond to all of the panels.

Figure 7 shows the enhancement in the LPR with the inclusion of single-environment structures for the representative low- and high-density a-C films. When 10 diamond-like single-environment structures are included, the LPR enhancement is mostly observed for the local environments in the high-density a-C film (Figure 7a, stronger green color). Conversely, when 10 graphite-like single-environment structures are included, the LPR enhancement takes place primarily for the environments in the low-density film (Figure 7b). For 100 local environments across both films that are the most similar to the newly added single-environment structures, we observe an average LPR enhancement of 31% for the diamond-like environments and 54% for the graphite-like environments. Interestingly, when both types of single-environment structures are incorporated into the training set, i.e., five diamond-like and five graphite-like single-environment structures, enhancement of the LPR is observed throughout both low- and high-density a-C films (Figure 7c), with an average enhancement of 36% for the 200 previously selected local environments. In terms of , inclusion of the single-environment structures reduces the uncertainty by up to 87%. Such improvements take place while the accuracy of the models remains largely the same, where the % RMSE on the test set changes from 12 to 14% at most.

These results prove that generation and inclusion of single-environment structures similar to the local environments of interest is a highly effective strategy to systematically enhance the LPR and improve the robustness in the local predictions of the ML model. It is striking to see that notable enhancement is already induced by replacing only 1% of the data set with single-environment structures. While only diamond- and graphite-like single-environment structures are considered here, the discovery and inclusion of other single-environment samples, diverse in their structures yet similar to the local environments of interest, would likely induce further enhancements in the LPR.

The case studies up to this point clearly demonstrate how the robustness in the local predictions can be estimated using the LPR. To provide a practical example in which an increase in the LPR is also associated with improved model transferability, we consider another case study on a-C, assessing the transferability of ML models trained on bulk, high-density a-C structures to the surface-containing a-C structures from Deringer et al.^61^ For a model trained exclusively on 1000 high-density (2.9–3.6 g/cm^3^) a-C structures, low LPR is observed for the surface atoms of the surface-containing structures (see Figure S17). For models that are modified by training on data sets where 1% is replaced with either graphite-like single-environment structures or low-density (<2.1 g/cm^3^) a-C structures, significant enhancement in the LPR for the surface atoms is observed. These two models show much higher accuracy in the predictions for the out-of-sample surface-containing structures (Figure S18). The RMSE on the total energy per atom decreases from 722 meV of the original model to 446 meV (introduction of single-environment structures) and 167 meV (introduction of low-density structures). This illustrates how striving for higher LPR and more robust local predictions can also lead to improved stability and transferability of the model in terms of global predictions. Further details of this case study can be found in the Supporting Information.

## 6 Extension to NN Models

Thus, far, we have applied the LPR analysis only to linear and kernel models, which are associated with a convex loss function that can be minimized analytically. We now extend our study to the case of NN models. NNs are a large class of regression methods in atomistic ML.^23,25−27,62−65^ They are generally regarded to be far more “flexible” than their linear counterparts, given the significantly larger number of weight parameters involved in training the model. One peculiarity of NN models is that they cannot be optimized in an analytical, deterministic way: model training is often carried out with recursive numerical methods and does not exactly reach the actual minimum, which is an assumption underlying the formulation of the LPR. Here, we assume that the NN models trained for our analysis are close enough to the minimum for the LPR formulations to still be applicable. Another point to note is that the second-order derivative  of eq 4 does not vanish in general for NN models. Nevertheless, as is customary in the context of nonlinear optimization,^66^ we assume a negligible statistical correlation between (*Y*_*A*_ – *Ỹ*_*A*_) and  over the training set and drop the second term on the right-hand side of eq 6. In practice, we obtain **H**^*o*^ by computing and accumulating  for the local environments in the training set by using the automatic differentiation framework in PyTorch.^67^

We train a simple multilayer perceptron model with 2 hidden layers, each composed of 16 nodes with a nonlinear sigmoid activation function. The model is trained on the same carbon data set as in the previous section with the SOAP power-spectrum vectors as the input layer and their local energies predicted at the output layer. We adopt the Behler–Parrinello approach of summing the local NN predictions outside of the NN model to regress global quantities.^23^ We also perform explicit L^2^ regularization of the NN model weights rather than the conventional early stopping with respect to a validation set to retain the loss function used in deriving the LPR and ensure comparability with the previous linear models.^68^ Full details of NN model training and LPR calculation are provided in the Supporting Information. The test set % RMSE for the resulting NN model is 12%.

**Figure 8:**
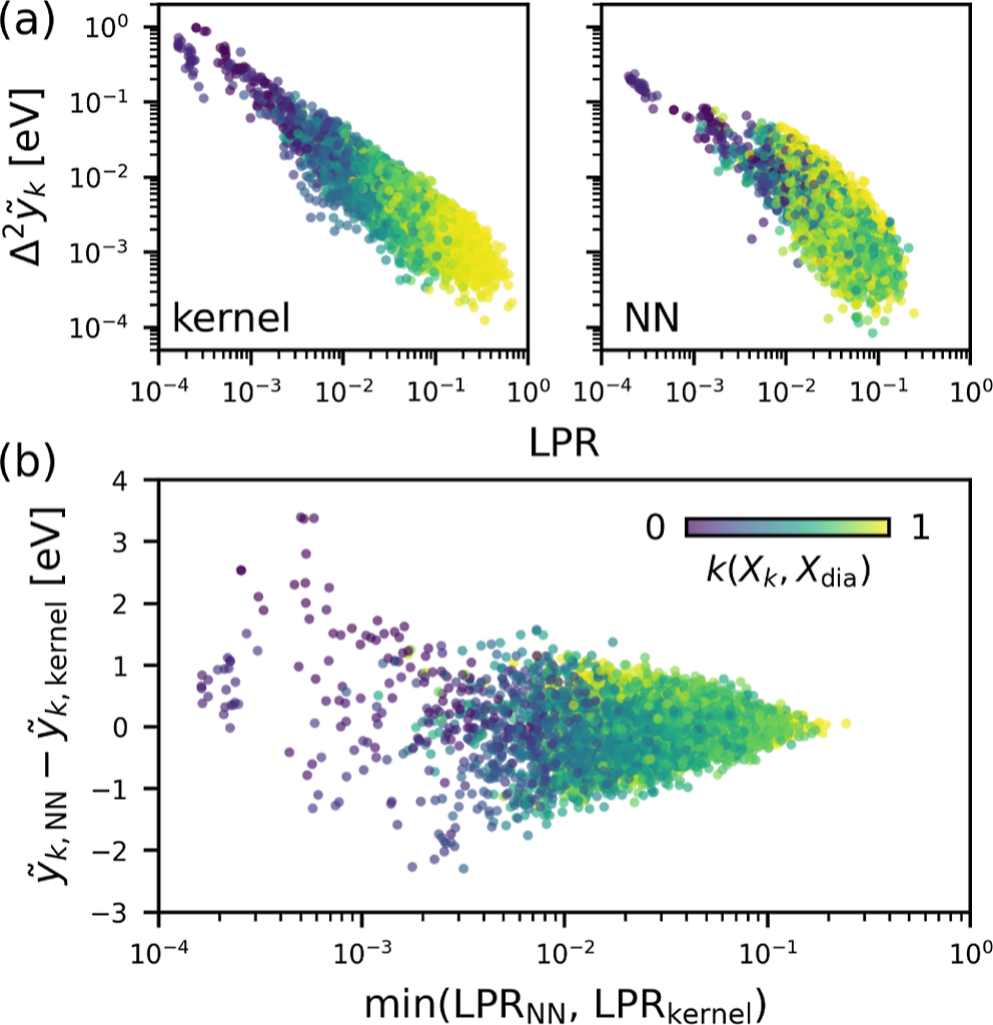
Extension of the LPR analysis to the NN model. (a) Δ^2^*ỹ*_*k*_ for a committee of 10 models vs the LPR, calculated for the low-density carbon film using the sparse kernel model (left) and NN model (right). (b) Difference in the local energy predictions vs minimum LPR between the sparse kernel model and the NN model. In all cases, data points are colored by the SOAP kernel similarity of the local environments to that of pristine diamond.

For the analysis, the LPR and Δ^2^*ỹ*_*k*_ of the low-density carbon film from the previous section are calculated for the sparse kernel model and the NN model. In Figure 8a, both models exhibit a clear inverse proportionality between the LPR and Δ^2^*ỹ*_*k*_ for the local predictions across a committee of models. This corroborates the relationship between the LPR and the uncertainty in the local predictions and how it also extends to nonlinear NN models. Additionally, in Figure 8b, the difference in the local energy predictions of the two models diminishes when the LPR increases. This provides a clear example of how the LPR can be used to quantify the stability of local predictions to the choice of ML architecture, legitimizing to an extent the use of atom-centered contributions for posthoc interpretations.

An interesting difference to be noted here is the correlation between the LPR (or Δ^2^*ỹ*_*k*_) and the local environment similarity to diamond. In the sparse kernel model, Δ^2^*ỹ*_*k*_ decreases with increasing similarity to diamond, which stems from the abundance of diamond-like environments in the training set (Figure S19). For the NN model, such a correlation is absent, and the lowest values of Δ^2^*ỹ*_*k*_ are also observed for the environments that differ substantially from diamond. This suggests that the heuristic observations of the direct dependence of the LPR on the data set composition, which we have seen for linear and kernel models, apply only partially to the NN model, which can be, in part, attributed to its nonlinearity (see Figure S20). Here, we note that the nonconvex nature of the NN optimization process, and the fact that the LPR is defined as a local response of the loss around a single local minimum, makes it difficult to compare the LPR between models that are independently trained on different data sets and to verify how much of the previously observed trends with respect to data set modifications apply to the NN models.

## 7 Conclusions

While the local decomposition approach commonly adopted by atomistic ML models has proven to be very successful, it inevitably introduces a degree of arbitrariness into the model predictions, which are made locally and without a well-defined target. While it is not possible to rigorously justify these atom-centered decompositions, one should still make them as robust as possible to the model training details, such as the model architecture and the training data set makeup. To this end, we have devised LPR, which allows one to quantify the robustness of the local predictions made by atomistic ML models. For a range of models and data sets, we have demonstrated that the LPR can vary drastically between different local environments. Local predictions of atomistic ML models should therefore be interpreted cautiously, and the LPR should be taken into consideration alongside the model predictions.

Our analyses have also shown that the process in which the LPR becomes determined for an ML model prediction is largely dependent on the degeneracies associated with the local decomposition of the target global quantities. To systematically improve the LPR, the data set for model training should be judiciously constructed to eliminate as much of the degeneracy as possible. For this, all local environments of interest should be sufficiently well-represented in the data set for model training. In cases where multiple atomic types or species are present, many different chemical and structural compositions must be probed by the data set to eliminate the degeneracy between the types or species. One can also generate and include single-environment structures to systematically enhance the LPR of a model for the local environments of particular interest. Last, the LPR can even be utilized as a metric of uncertainty across different types of atomistic ML models.

The clear connection between the LPR and uncertainty suggests that measures of error in the local predictions, which are readily available in several widely used models, can be used to compute a substitute for the LPR. This makes it possible for one to easily expand on the insights found in our study for a wider range of atomistic ML models. As the derivation of LPR is not limited to the atomic decomposition primarily dealt with in this study, it can be extended to other decomposition schemes: multiple body-order decomposition, short-range versus long-range decomposition, and so forth. This allows one to precisely identify where the ML model lacks robustness in the predictions and to identify effective ways to improve it.

## Data Availability

The data that support the findings of this study and analysis scripts employed to generate the plots and relevant results are available on the Materials Cloud platform.^68^ See DOI: 10.24435/materialscloud:re-0d.

## Supporting Information Available

Details of data set construction and model training for all the atomistic ML models; details of single-environment structure generation; case studies with linear ridge regression models trained on the demonstrative chemical data sets; a-C data set composition; and discussion of observed trends in the LPR (PDF)

## Footnotes

1 In the projected process approximation, a further term arises, , which vanishes in the SR approximation due to the Nyström approximation for the covariance function. See Chapter 8 of ref (56)

2 Here we note that there is an exception to this, which is when *x*_*k*_ is the feature of group *X*_*A*_ in the training set where *X*_*A*_ = [*N*_*A*_*x*_*A*_]. In real chemical systems, this corresponds to the case of single-environment structures discussed later on.

3 The value of 2.7 Å is smaller than the commonly used value of 2.85 Å for silicon bond detection. This value was heuristically chosen to prevent the neighboring silicon atoms from being present in the distance range of the smooth cutoff function of the SOAP descriptor.

4 Similar to silicon, the smaller value of 1.82 Å as opposed to 1.85 Å was heuristically chosen to prevent the neighboring carbon atoms from being present in the distance range of the smooth cutoff function of the SOAP descriptor.

## References

[ref1] Prodan E.; Kohn W. Nearsightedness of Electronic Matter. Proc. Natl. Acad. Sci. U.S.A. 2005, 102, 11635–11638. 10.1073/pnas.0505436102. 16087868 PMC1188007

[ref2] Yang W. Direct Calculation of Electron Density in Density-Functional Theory. Phys. Rev. Lett. 1991, 66, 1438–1441. 10.1103/PhysRevLett.66.1438. 10043209

[ref3] Kohn W. Density Functional and Density Matrix Method Scaling Linearly with the Number of Atoms. Phys. Rev. Lett. 1996, 76, 3168–3171. 10.1103/PhysRevLett.76.3168. 10060892

[ref4] White C. A.; Johnson B. G.; Gill P. M.; Head-Gordon M. Linear scaling density functional calculations via the continuous fast multipole method. Chem. Phys. Lett. 1996, 253, 268–278. 10.1016/0009-2614(96)00175-3.

[ref5] Baer R.; Head-Gordon M. Chebyshev Expansion Methods for Electronic Structure Calculations on Large Molecular Systems. J. Chem. Phys. 1997, 107, 10003–10013. 10.1063/1.474158.

[ref6] Ochsenfeld C.; White C. A.; Head-Gordon M. Linear and sublinear scaling formation of Hartree–Fock-type exchange matrices. J. Chem. Phys. 1998, 109, 1663–1669. 10.1063/1.476741.

[ref7] Goedecker S. Linear scaling electronic structure methods. Rev. Mod. Phys. 1999, 71, 1085–1123. 10.1103/RevModPhys.71.1085.

[ref8] Liang W. Z.; Saravanan C.; Shao Y.; Baer R.; Bell A. T.; Head-Gordon M. Improved Fermi Operator Expansion Methods for Fast Electronic Structure Calculations. J. Chem. Phys. 2003, 119, 4117–4125. 10.1063/1.1590632.

[ref9] Saravanan C.; Shao Y.; Baer R.; Ross P. N.; Head–Gordon M. Sparse matrix multiplications for linear scaling electronic structure calculations in an atom-centered basis set using multiatom blocks. J. Comput. Chem. 2003, 24, 618–622. 10.1002/jcc.10224. 12632476

[ref10] Sodt A.; Subotnik J. E.; Head-Gordon M. Linear scaling density fitting. J. Chem. Phys. 2006, 125, 194109 10.1063/1.2370949. 17129091

[ref11] Womack J. C.; Mardirossian N.; Head-Gordon M.; Skylaris C.-K. Self-consistent implementation of meta-GGA functionals for the ONETEP linear-scaling electronic structure package. J. Chem. Phys. 2016, 145, 204114 10.1063/1.4967960. 27908114

[ref12] Dronskowski R.; Bloechl P. E. Crystal Orbital Hamilton Populations (COHP): Energy-resolved visualization of chemical bonding in solids based on density-functional calculations. J. Phys. Chem. 1993, 97, 8617–8624. 10.1021/j100135a014.

[ref13] Amadon B.; Lechermann F.; Georges A.; Jollet F.; Wehling T. O.; Lichtenstein A. I. Plane-wave based electronic structure calculations for correlated materials using dynamical mean-field theory and projected local orbitals. Phys. Rev. B 2008, 77, 205112 10.1103/PhysRevB.77.205112.

[ref14] Szalewicz K. Symmetry-adapted perturbation theory of intermolecular forces. Wiley Interdiscip. Rev.: Comput. Mol. Sci. 2012, 2, 254–272. 10.1002/wcms.86.

[ref15] Maintz S.; Deringer V. L.; Tchougréeff A. L.; Dronskowski R. LOBSTER: A tool to extract chemical bonding from plane-wave based DFT. J. Comput. Chem. 2016, 37, 1030–1035. 10.1002/jcc.24300. 26914535 PMC5067632

[ref16] Nelson R.; Ertural C.; George J.; Deringer V. L.; Hautier G.; Dronskowski R. LOBSTER: Local orbital projections, atomic charges, and chemical-bonding analysis from projector-augmented-wave-based density-functional theory. J. Comput. Chem. 2020, 41, 1931–1940. 10.1002/jcc.26353. 32531113

[ref17] George J.; Petretto G.; Naik A.; Esters M.; Jackson A. J.; Nelson R.; Dronskowski R.; Rignanese G.-M.; Hautier G. Automated Bonding Analysis with Crystal Orbital Hamilton Populations. ChemPlusChem 2022, 87, e202200123 10.1002/cplu.202200123. 35762686

[ref18] Fox S. J.; Pittock C.; Fox T.; Tautermann C. S.; Malcolm N.; Skylaris C.-K. Electrostatic embedding in large-scale first principles quantum mechanical calculations on biomolecules. J. Chem. Phys. 2011, 135, 224107 10.1063/1.3665893. 22168680

[ref19] Lever G.; Cole D. J.; Lonsdale R.; Ranaghan K. E.; Wales D. J.; Mulholland A. J.; Skylaris C.-K.; Payne M. C. Large-Scale Density Functional Theory Transition State Searching in Enzymes. J. Phys. Chem. Lett. 2014, 5, 3614–3619. 10.1021/jz5018703. 26278727

[ref20] Yun S.; Zhou X.; Even J.; Hagfeldt A. Theoretical Treatment of CH_3_NH_3_PbI_3_ Perovskite Solar Cells. Angew. Chem., Int. Ed. 2017, 56, 15806–15817. 10.1002/anie.201702660. 28544169

[ref21] Skorupskii G.; Trump B. A.; Kasel T. W.; Brown C. M.; Hendon C. H.; Dincă M. Efficient and tunable one-dimensional charge transport in layered lanthanide metal–organic frameworks. Nat. Chem. 2019, 12, 131–136. 10.1038/s41557-019-0372-0. 31767997 PMC11060427

[ref22] Chong S.; Kim J. Rational modifications of PCN-700 to induce electrical conductivity: a computational study. Dalton Trans. 2020, 49, 102–113. 10.1039/C9DT03865E. 31793579

[ref23] Behler J.; Parrinello M. Generalized Neural-Network Representation of High-Dimensional Potential-Energy Surfaces. Phys. Rev. Lett. 2007, 98, 146401 10.1103/PhysRevLett.98.146401. 17501293

[ref24] Bartók A. P.; Payne M. C.; Kondor R.; Csányi G. Gaussian Approximation Potentials: The Accuracy of Quantum Mechanics, without the Electrons. Phys. Rev. Lett. 2010, 104, 136403 10.1103/PhysRevLett.104.136403. 20481899

[ref25] Smith J. S.; Isayev O.; Roitberg A. E. ANI-1: An Extensible Neural Network Potential with DFT Accuracy at Force Field Computational Cost. Chem. Sci. 2017, 8, 3192–3203. 10.1039/C6SC05720A. 28507695 PMC5414547

[ref26] Schütt K. T.; Sauceda H. E.; Kindermans P.-J.; Tkatchenko A.; Müller K.-R. SchNet – A Deep Learning Architecture for Molecules and Materials. J. Chem. Phys. 2018, 148, 241722. 29960322 10.1063/1.5019779

[ref27] Ko T. W.; Finkler J. A.; Goedecker S.; Behler J. A fourth-generation high-dimensional neural network potential with accurate electrostatics including non-local charge transfer. Nat. Commun. 2021, 12, 398 10.1038/s41467-020-20427-2. 33452239 PMC7811002

[ref28] Artrith N.; Behler J. High-Dimensional Neural Network Potentials for Metal Surfaces: A Prototype Study for Copper. Phys. Rev. B 2012, 85, 045439 10.1103/PhysRevB.85.045439.

[ref29] Sosso G. C.; Miceli G.; Caravati S.; Behler J.; Bernasconi M. Neural Network Interatomic Potential for the Phase Change Material GeTe. Phys. Rev. B 2012, 85, 174103 10.1103/PhysRevB.85.174103.

[ref30] Deringer V. L.; Csányi G. Machine Learning Based Interatomic Potential for Amorphous Carbon. Phys. Rev. B 2017, 95, 094203 10.1103/PhysRevB.95.094203.

[ref31] Bartók A. P.; Kermode J.; Bernstein N.; Csányi G. Machine Learning a General-Purpose Interatomic Potential for Silicon. Phys. Rev. X 2018, 8, 041048 10.1103/PhysRevX.8.041048.

[ref32] Lopanitsyna N.; Fraux G.; Springer M. A.; De S.; Ceriotti M. Modeling high-entropy transition metal alloys with alchemical compression. Phys. Rev. Mater. 2023, 7, 045802 10.1103/PhysRevMaterials.7.045802.

[ref33] Sosso G. C.; Donadio D.; Caravati S.; Behler J.; Bernasconi M. Thermal transport in phase-change materials from atomistic simulations. Phys. Rev. B 2012, 86, 104301 10.1103/PhysRevB.86.104301.

[ref34] Verdi C.; Karsai F.; Liu P.; Jinnouchi R.; Kresse G. Thermal transport and phase transitions of zirconia by on-the-fly machine-learned interatomic potentials. npj Comput. Mater. 2021, 7, 156 10.1038/s41524-021-00630-5.

[ref35] Deng J.; Stixrude L. Thermal conductivity of silicate liquid determined by machine learning potentials. Geophys. Res. Lett. 2021, 48, e2021GL093806 10.1029/2021gl093806.

[ref36] Tisi D.; Zhang L.; Bertossa R.; Wang H.; Car R.; Baroni S. Heat transport in liquid water from first-principles and deep neural network simulations. Phys. Rev. B 2021, 104, 224202 10.1103/PhysRevB.104.224202.

[ref37] Pegolo P.; Baroni S.; Grasselli F. Temperature-and vacancy-concentration-dependence of heat transport in Li_3_ClO from multi-method numerical simulations. npj Comput. Mater. 2022, 8, 24 10.1038/s41524-021-00693-4.

[ref38] Brorsson J.; Hashemi A.; Fan Z.; Fransson E.; Eriksson F.; Ala-Nissila T.; Krasheninnikov A. V.; Komsa H.-P.; Erhart P. Efficient Calculation of the Lattice Thermal Conductivity by Atomistic Simulations with Ab Initio Accuracy. Adv. Theory Simul. 2022, 5, 2100217 10.1002/adts.202100217.

[ref39] Langer M. F.; Knoop F.; Carbogno C.; Scheffler M.; Rupp M. Heat flux for semi-local machine-learning potentials. arXiv preprint arXiv:2303.14434 2023,.

[ref40] Irving J. H.; Kirkwood J. G. The statistical mechanical theory of transport processes. IV. The equations of hydrodynamics. J. Chem. Phys. 1950, 18, 817–829. 10.1063/1.1747782.

[ref41] Ben Mahmoud C.; Anelli A.; Csányi G.; Ceriotti M. Learning the electronic density of states in condensed matter. Phys. Rev. B 2020, 102, 235130 10.1103/PhysRevB.102.235130.

[ref42] Deringer V. L.; Bernstein N.; Csányi G.; Ben Mahmoud C.; Ceriotti M.; Wilson M.; Drabold D. A.; Elliott S. R. Origins of Structural and Electronic Transitions in Disordered Silicon. Nature 2021, 589, 59–64. 10.1038/s41586-020-03072-z. 33408379

[ref43] Ellis J. A.; Fiedler L.; Popoola G. A.; Modine N. A.; Stephens J. A.; Thompson A. P.; Cangi A.; Rajamanickam S. Accelerating finite-temperature Kohn-Sham density functional theory with deep neural networks. Phys. Rev. B 2021, 104, 035120 10.1103/PhysRevB.104.035120.

[ref44] Lopanitsyna N.; Ben Mahmoud C.; Ceriotti M. Finite-Temperature Materials Modeling from the Quantum Nuclei to the Hot Electron Regime. Phys. Rev. Mater. 2021, 5, 043802 10.1103/PhysRevMaterials.5.043802.

[ref45] Ben Mahmoud C.; Grasselli F.; Ceriotti M. Predicting hot-electron free energies from ground-state data. Phys. Rev. B 2022, 106, L121116 10.1103/physrevb.106.l121116.

[ref46] Schütt K. T.; Arbabzadah F.; Chmiela S.; Müller K. R.; Tkatchenko A. Quantum-Chemical Insights from Deep Tensor Neural Networks. Nat. Commun. 2017, 8, 13890 10.1038/ncomms13890. 28067221 PMC5228054

[ref47] Deringer V. L.; Pickard C. J.; Csányi G. Data-Driven Learning of Total and Local Energies in Elemental Boron. Phys. Rev. Lett. 2018, 120, 156001 10.1103/PhysRevLett.120.156001. 29756876

[ref48] Bernstein N.; Bhattarai B.; Csányi G.; Drabold D. A.; Elliott S. R.; Deringer V. L. Quantifying Chemical Structure and Machine-Learned Atomic Energies in Amorphous and Liquid Silicon. Angew. Chem., Int. Ed. 2019, 58, 7057–7061. 10.1002/anie.201902625. PMC6563111 30835962

[ref49] Cersonsky R. K.; Pakhnova M.; Engel E. A.; Ceriotti M. A data-driven interpretation of the stability of organic molecular crystals. Chem. Sci. 2023, 14, 1272–1285. 10.1039/D2SC06198H. 36756329 PMC9891366

[ref50] Wang S.; Liu Y.; Mo Y. Frustration in Super-Ionic Conductors Unraveled by the Density of Atomistic States. Angew. Chem., Int. Ed. 2023, 62, e202215544 10.1002/anie.202215544. 36749663

[ref51] El-Machachi Z.; Wilson M.; Deringer V. L. Exploring the configurational space of amorphous graphene with machine-learned atomic energies. Chem. Sci. 2022, 13, 13720–13731. 10.1039/D2SC04326B. 36544732 PMC9710228

[ref52] Gardner J. L. A.; Faure Beaulieu Z.; Deringer V. L. Synthetic data enable experiments in atomistic machine learning. Digital Discovery 2023, 2, 651–662. 10.1039/D2DD00137C.

[ref53] Eckhoff M.; Behler J. From Molecular Fragments to the Bulk: Development of a Neural Network Potential for MOF-5. J. Chem. Theory Comput. 2019, 15, 3793–3809. 10.1021/acs.jctc.8b01288. 31091097

[ref54] Zhai Y.; Caruso A.; Bore S. L.; Luo Z.; Paesani F. A “short blanket” dilemma for a state-of-the-art neural network potential for water: Reproducing experimental properties or the physics of the underlying many-body interactions?. J. Chem. Phys. 2023, 158, 084111 10.1063/5.0142843. 36859071

[ref55] Deringer V. L.; Bartók A. P.; Bernstein N.; Wilkins D. M.; Ceriotti M.; Csányi G. Gaussian Process Regression for Materials and Molecules. Chem. Rev. 2021, 121, 10073–10141. 10.1021/acs.chemrev.1c00022. 34398616 PMC8391963

[ref56] Rasmussen C. E. Gaussian Processes for Machine Learning; MIT Press, 2006;.

[ref57] Bartók A. P.; Kondor R.; Csányi G. On Representing Chemical Environments. Phys. Rev. B 2013, 87, 184115 10.1103/PhysRevB.87.184115.

[ref58] Caro M. A.; Csányi G.; Laurila T.; Deringer V. L. Machine Learning Driven Simulated Deposition of Carbon Films: From Low-Density to Diamondlike Amorphous Carbon. Phys. Rev. B 2020, 102, 174201 10.1103/PhysRevB.102.174201.

[ref59] Stukowski A. Visualization and analysis of atomistic simulation data with OVITO–the Open Visualization Tool. Modell. Simul. Mater. Sci. Eng. 2010, 18, 015012 10.1088/0965-0393/18/1/015012.

[ref60] Caro M. A.; Deringer V. L.; Koskinen J.; Laurila T.; Csányi G. Growth Mechanism and Origin of High *sp*^3^ Content in Tetrahedral Amorphous Carbon. Phys. Rev. Lett. 2018, 120, 166101 10.1103/PhysRevLett.120.166101. 29756912

[ref61] Deringer V. L.; Caro M. A.; Jana R.; Aarva A.; Elliott S. R.; Laurila T.; Csányi G.; Pastewka L. Computational Surface Chemistry of Tetrahedral Amorphous Carbon by Combining Machine Learning and Density Functional Theory. Chem. Mater. 2018, 30, 7438–7445. 10.1021/acs.chemmater.8b02410.

[ref62] Batatia I.; Kovacs D. P.; Simm G.; Ortner C.; Csanyi G. MACE: Higher Order Equivariant Message Passing Neural Networks for Fast and Accurate Force Fields. Adv. Neural Inf. Process 2022, 35, 11423–11436.

[ref63] Batzner S.; Musaelian A.; Sun L.; Geiger M.; Mailoa J. P.; Kornbluth M.; Molinari N.; Smidt T. E.; Kozinsky B. E(3)-equivariant graph neural networks for data-efficient and accurate interatomic potentials. Nat. Commun. 2022, 13, 2453 10.1038/s41467-022-29939-5. 35508450 PMC9068614

[ref64] Musaelian A.; Batzner S.; Johansson A.; Sun L.; Owen C. J.; Kornbluth M.; Kozinsky B. Learning local equivariant representations for large-scale atomistic dynamics. Nat. Commun. 2023, 14, 579 10.1038/s41467-023-36329-y. 36737620 PMC9898554

[ref65] Pozdnyakov S. N.; Ceriotti M. Smooth, exact rotational symmetrization for deep learning on point clouds. arXiv preprint arXiv:2305.19302 2023,.

[ref66] Press W. H.; Teukolsky S. A.; Vetterling W. T.; Flannery B. P. Numerical recipes. The art of scientific computing, 3rd ed.; Cambridge University Press, 2007; pp 800–801.

[ref67] Paszke A.; Gross S.; Massa F.; Lerer A.; Bradbury J.; Chanan G.; Killeen T.; Lin Z.; Gimelshein N.; Antiga L.; Desmaison A.; Kopf A.; Yang E.; DeVito Z.; Raison M.; Tejani A.; Chilamkurthy S.; Steiner B.; Fang L.; Bai J.; Chintala S. PyTorch: An Imperative Style, High-Performance Deep Learning Library. Adv. Neural Inf. Process 2019, 32, 8024–8035.

[ref68] Talirz L.; Kumbhar S.; Passaro E.; Yakutovich A. V.; Granata V.; Gargiulo F.; Borelli M.; Uhrin M.; Huber S. P.; Zoupanos S.; Adorf C. S.; Andersen C. W.; Schütt O.; Pignedoli C. A.; Passerone D.; VandeVondele J.; Schulthess T. C.; Smit B.; Pizzi G.; Marzari N. Materials Cloud, a Platform for Open Computational Science. Sci. Data 2020, 7, 299 10.1038/s41597-020-00637-5. 32901046 PMC7479138

