# Supplementary material for: Robustness of Local Predictions in Atomistic Machine Learning Models
Source: J Chem Theory Comput. 2023 Nov 10;19(22):8020–31. doi: 10.1021/acs.jctc.3c00704 (PMC10688186; doi:10.1021/acs.jctc.3c00704)
Supplement: Supplementary file 1 — ct3c00704_si_001.pdf [file ct3c00704_si_001.pdf]

Supporting Information for "Robustness of Local Predictions in Atomistic Machine Learning Models"

Sanggyu Chong[1], Federico Grasselli[1], Chiheb Ben Mahmoud[1], Joe D. Morrow[2], Volker L. Deringer[2], and Michele Ceriotti[1,*]

[1]Laboratory of Computational Science and Modeling, Institute of Materials, École Polytechnique Fédérale de Lausanne, 1015 Lausanne, Switzerland
[2]Department of Chemistry, Inorganic Chemistry Laboratory, University of Oxford, Oxford OX1 3QR, United Kingdom
[*]E-mail: michele.ceriotti@epfl.ch

Contents

1 Supplementary Figures for "Proof-of-Concept Using Toy Models" 2

2 Details of ML Model Training for "Case Studies on Real Chemical Systems" 3

3 Supplementary Figures for "Case Studies on Demonstrative Chemical Datasets" 5

4 Linear Ridge Regression Model Results for "Case Studies on Real Chemical Systems" 10

5 Details of ML Model Training for "More Realistic Applications" 19

6 Details of ML Model Training for "Extension to Neural Network Models" 24

7 Supplementary Figures for "Extension to Neural Network Models" 25

1 Supplementary Figures for "Proof-of-Concept Using Toy Models"

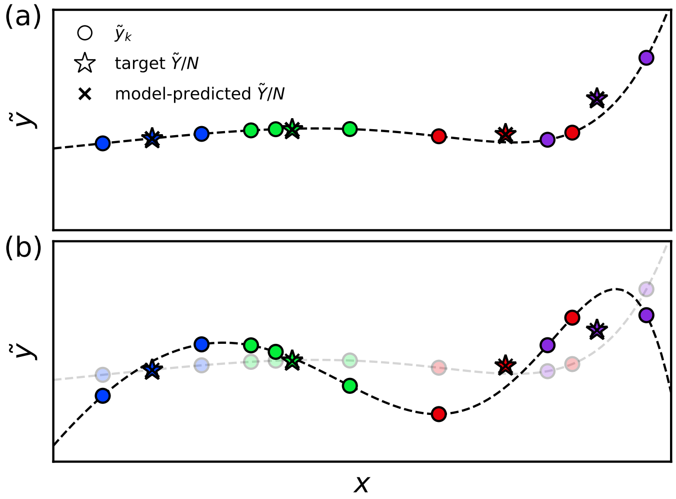

Figure S1: Toy model that makes predictions $\tilde{y}$ for a scalar input x. Model training was performed using a set of global quantities that sum together contributions from multiple input values. The model, $\tilde{y}(x)$, is shown with a dashed line. Local predictions for x_k that appear in the training set are plotted with circles, colored according to the group X_A that it contributes to. Star-shaped markers correspond to Y_A/N of the target global quantities. The crosses show the corresponding predictions of the model, i.e. $\sum \tilde{y}_k/N$. (a) The original toy model and its local and global predictions. (b) Another model trained by perturbing an arbitrary $\tilde{y}_k$ using the Lagrange multiplier approach. The model makes vastly different local predictions from the original model, yet still retains the same predictions for the global quantities.

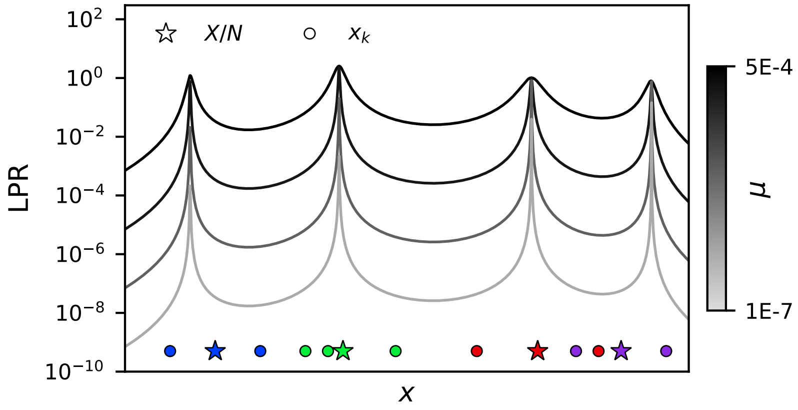

Figure S2: The LPR profile across the entire x range of interest, for several different values of the regularizer strength μ. The positions of local features x_k belonging to different groups X_A that comprise the training set are presented at the bottom in circles, along with the average local feature X/N for each of the groups in stars.

2 Details of ML Model Training for "Case Studies on Real Chemical Systems"

In all of the case studies, sparse kernel models[1] were trained to predict the total energies per atom of the chemical structures by taking the average of the local energy contributions of the constituent atoms. The averaging of the target and the prediction was done to ensure that each structure receives the same regularization strength in cases where training set contains structures with different number of atoms. The local environments of the atoms were represented using the Smooth Overlap of Atomic Positions (SOAP) feature vectors, using rascaline, and the Shifted cosine was used as the primary cutoff function, and in some cases, radial scaling of Willatt et al.[2] was additionally employed. SOAP similarity kernels with $\zeta = 2$ were used in all cases.

Dataset for the amorphous silicon (a-Si) used for ML model training was generated by running melt-quench molecular dynamics (MD) simulations of 64 atoms using the elemental silicon potential developed by Bartok et al.[3], and following the variable-quench rate protocol of Deringer et al.[4] Melting was performed at 2500 K for 20 ps, before quenching mostly at 10^{13} K/s down to 500K, except for slower cooling of 10^{12} K/s over the temperature range of the liquid to amorphous transition between 1250–1050 K. Structures that were, stochastically, entirely free of coordination defects as defined by a 2.85 Å radial cutoff were first selected — approximately 15% of an initial 12,000 -– and taken forward for training. Further refinement was performed to sample 500 structures where the atoms are all 4-coordinated, as determined by a new distance cutoff of 2.7 Å, which prevents the neighboring atoms from being present within the smooth cutoff function. Then, 50 structures that contain a varying number of under- or over-coordinated Si atoms were randomly chosen. The set of hyperparameters used in computing the feature vectors is shared in Table S1, where all of the parameters are kept the same as the model used to generate the training set structures except for the cutoff radius and cutoff width. The cutoff radius of 2.85 Å was chosen as it is the distance that exclusively focuses the local environment description on the first "shell" of neighbors, in which the targeted coordination effects are present. We note that normalization of the feature vectors was not performed to best capture the differences in the magnitude of the feature vectors that arise from a Si atom being under- or over-coordinated. In choosing the reference environments for sparse kernel model training, Farthest point sampling (FPS) method was used to choose the most diverse set of 500 local environments from the 500 structure dataset. Grid search on the regularization strength was performed to obtain the value that minimizes the RMSE on the validation set of 100 structures separately sampled. As explained in the main text, two separate models were trained, one using the original dataset with structures only containing the 4-coordinated Si atoms, and another where 10% of the structures are replaced with those containing under- or over-coordinated Si atoms. Both models achieved test set RMSEs of 0.004 eV.

Dataset of amorphous carbon (a-C) was also generated by running melt-quench MD simulations of 64 atoms using the elemental carbon potential of Deringer et al.[5]. Melting was done at 9000 K for 4 ps, before quenching to 300 K over 10 ps in the NVT ensemble. In the simulations, density was fixed so as to achieve the desired ratio of sp^2 to sp^3 atoms with maximum likelihood, which was found by scanning a range of densities to be 0.1435, 0.1517, and 0.1359 atoms/Å^3 for the 50:50 ratio, sp^2-rich, and sp^3-rich structures, respectively. From the trajectories from all of the simulations at different densities, 500 structures with a 1:1 ratio between the 3-coordinated and 4-coordinated (herein denoted as "sp^2" and "sp^3") environments, as detected by a bonding distance cutoff of 1.82 Å, were randomly sampled. Then, 50 structures with a significantly different ratios between sp^2 and sp^3 environments were also sampled. The set of SOAP hyperparameters used for carbon is also shared in Table S1, where, again, all of the parameters are kept the same as the model used for the MD simulation except for the cutoff radius, which was modified to exclusively capture the first shell of neighbors. For a-C as well, normalization of the feature vectors was not performed. FPS method was used to select 500 diverse reference environments from the 1:1 ratio dataset, and grid search was performed to choose the regularization strength that minimizes the RMSE on a separate validation set of 100 structures. Three models were trained, one using the original dataset with the structures exhibiting 1:1 ratio between sp^2 and sp^3, another where 10% of the structures are replaced with structures of different ratios, and a final one where one structure of the training set is replaced with crystalline diamond (structure taken from Materials Project, reference code mp-66). The three models achieved test set RMSEs of 0.018 eV, 0.017 eV, and 0.018 eV, respectively.

Dataset of gallium arsenide (GaAs) was acquired from a dataset used in a previous study by Imbalzano and Ceriotti.[6, 7] From the original dataset, 400 structures with a 1:1 ratio between Ga and As in the system were randomly selected. 40 structures with a different Ga:As ratio, 40 pure Ga, and 40 pure As structures were then randomly sampled and kept separately. The set of SOAP hyperparameters used for the case study are shared in Table S1, which were adopted from Lopanitsyna et al.[8], a study which successfully constructed an alchemical ML potential for 25 different elements. The SOAP descriptors were composed in a manner where the descriptors for Ga and As are strictly separated, and hence two separate models for Ga and As were effectively being trained. FPS method was used to select 400 reference environments, 200 of which were Ga and the other 200 As. Grid search was performed to find the regularization strength that minimizes the RMSE on a validation set of 100 structures. Four different models were trained, one using the original dataset with structures exhibiting 1:1 ratio of Ga and As, second one where 10% of the structures are replaced with structures of different Ga:As ratios, third one where 10% of the structures are replaced with pure Ga structures, and final one where 10% of the structures are replaced with pure As structures. The four models achieved test set RMSEs of 0.008 eV, 0.008 eV, 0.007 eV, and 0.007 eV, respectively.

**Dataset**	**a-Si**	**a-C**	**GaAs**
Interaction cutoff (Å)	2.85	1.85	5.0
Max radial	10	8	8
Max angular	12	8	4
Gaussian width (Å)	0.5	0.5	0.3
Cutoff width (Å)	0.1	0.1	0.5
Radial scaling	None	None	Willatt et al.[2]<br>scale = 2.0<br>rate = 0.8<br>exp = 2

Table S1: Table of SOAP hyperparameters used for our analyses presented in Section 2.

3 Supplementary Figures for "Case Studies on Demonstrative Chemical Datasets"

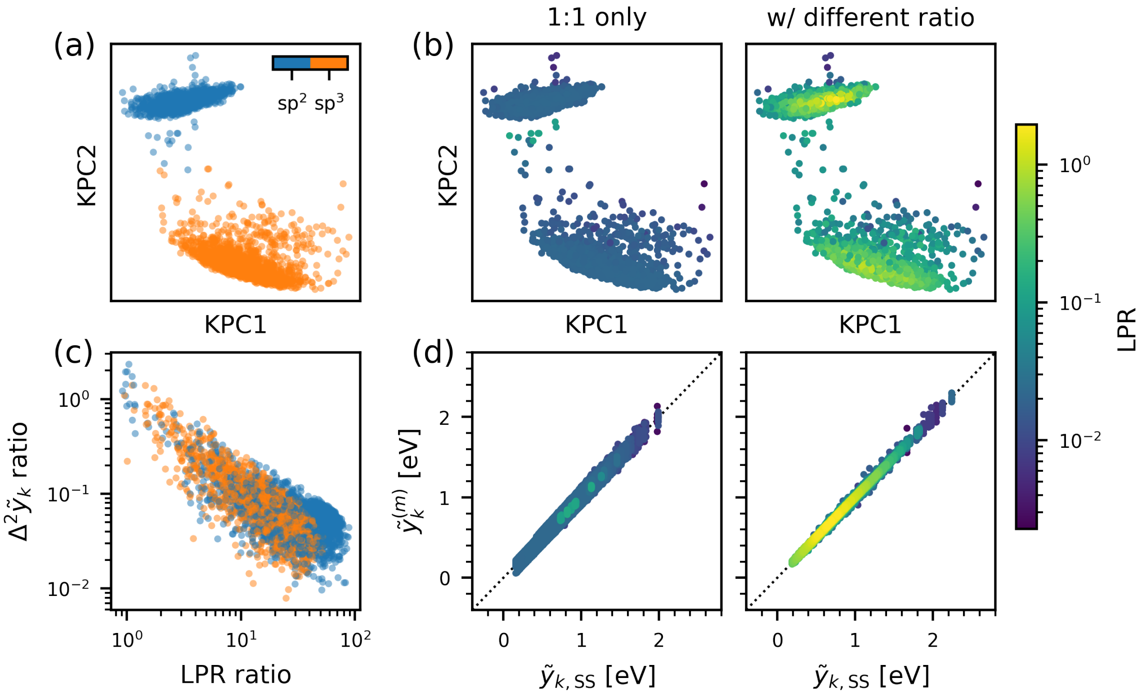

Figure S3: LPR and local energy predictions of models trained on the amorphous carbon (a-C) dataset before and after the inclusion of structures with a different ratio between sp^2 and sp^3 environments. (a) shows the kernel principal component analysis (KPCA) map with the points colored by of the atomic environments, where each point corresponds to an atomic environment in the test set of defect-containing a-Si structures. (b) shows the KPCA map colored by the LPR value from each model. (c) shows the ratio in the variance of the committee-predicted local energies ($\Delta^2 \tilde{y}_k$) vs. ratio in the LPR, before and after inclusion of the new structures. (d) shows the parity plots of the local energies predicted by a committee of 10 models vs. the committee average prediction, where the points are colored by the corresponding LPR values. Energy values are reported with respect to the atomic energy of diamond.

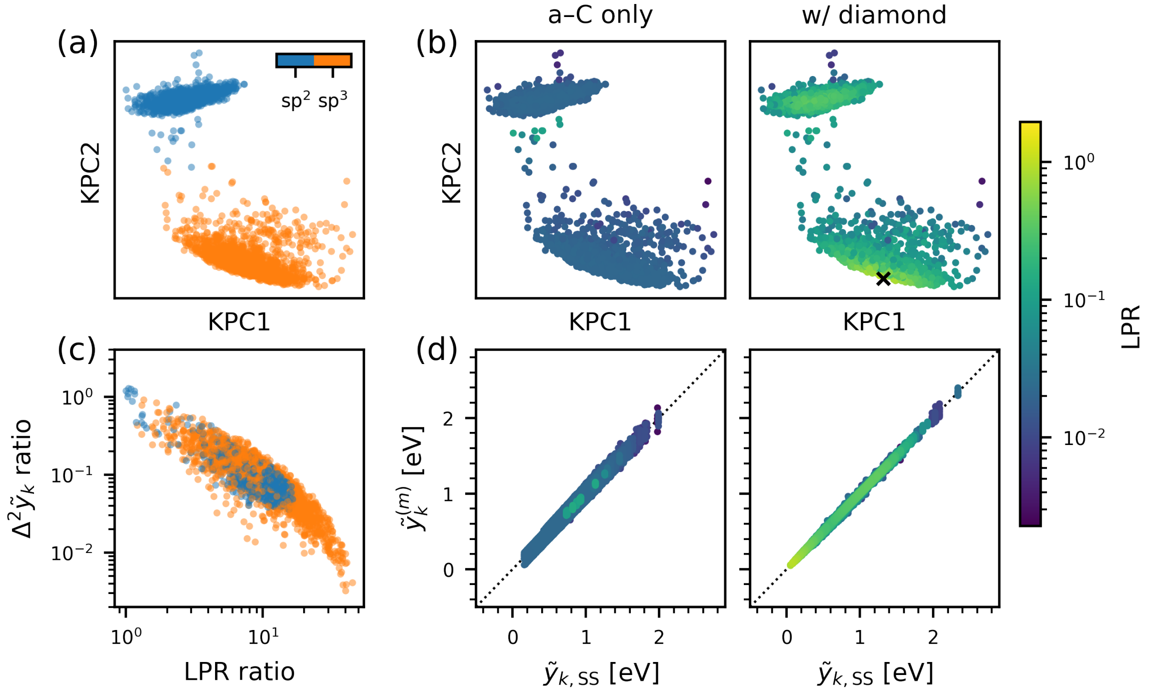

Figure S4: LPR and local energy predictions of models trained on the amorphous carbon (a-C) dataset before and after the inclusion of diamond. (a) shows the kernel principal component analysis (KPCA) map with the points colored by of the atomic environments, where each point corresponds to an atomic environment in the test set of defect-containing a-Si structures. (b) shows the KPCA map colored by the LPR value from each model. Position of diamond is marked with a cross. (c) shows the ratio in the variance of the committee-predicted local energies ($\Delta^2\tilde{y}_k$) vs. ratio in the LPR, before and after inclusion of diamond. (d) shows the parity plots of the local energies predicted by a committee of 10 models vs. the committee average prediction, where the points are colored by the corresponding LPR values. Energy values are reported with respect to the atomic energy of diamond.

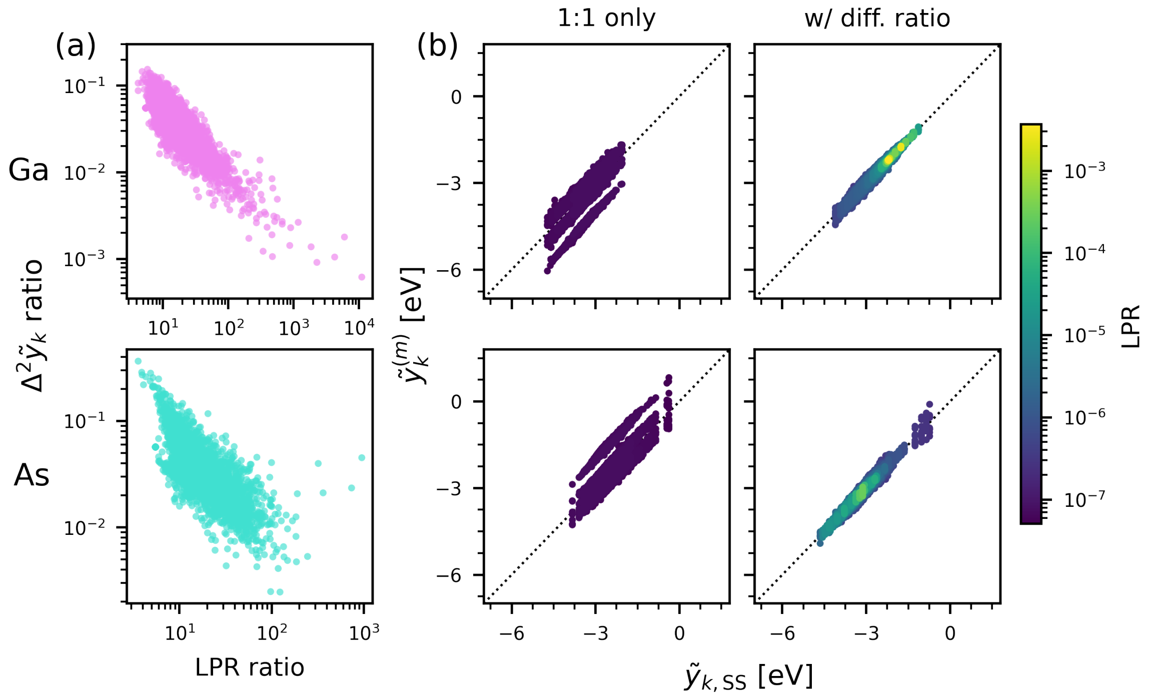

Figure S5: LPR and local energy predictions of models trained on the gallium arsenide (GaAs) dataset, before and after the inclusion of structures with a different Ga:As ratio. (a) shows the ratio in the variance of the committee-predicted local energies ($\Delta^2\tilde{y}_k$) vs. ratio in the LPR, before and after inclusion of the new structures. (b) shows the parity plots of the local energies predicted by a committee of 10 models vs. the committee average prediction, where the points are colored by the corresponding LPR values. Energy values are reported with respect to the single ion energies of Ga and As.

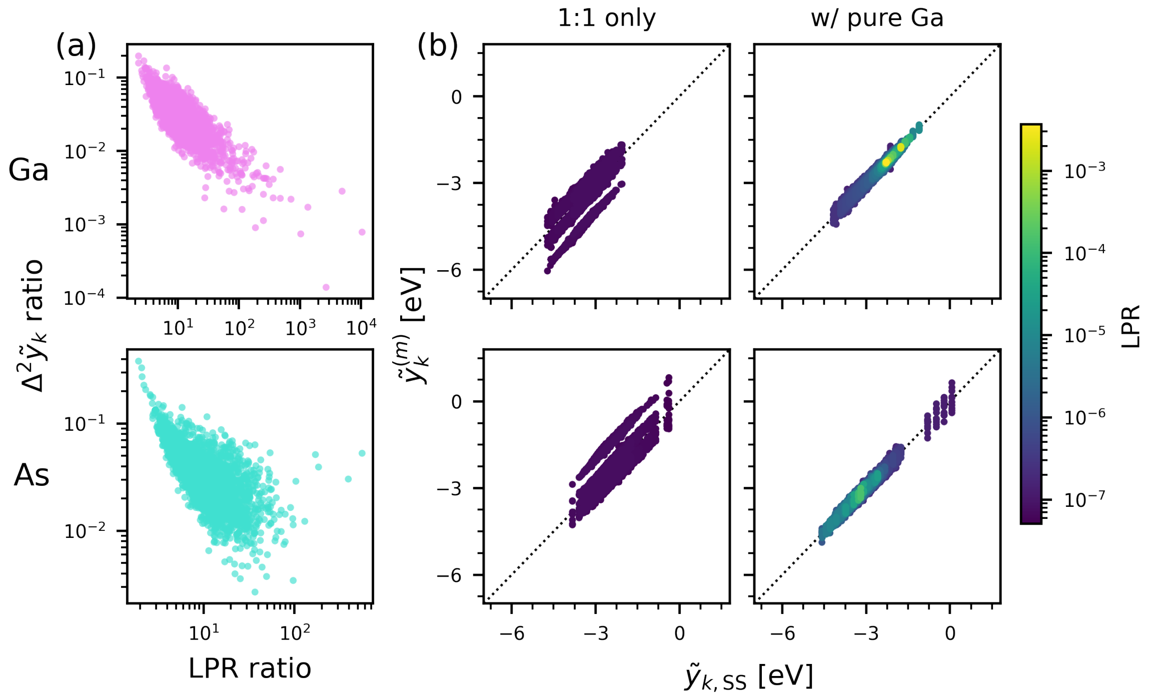

Figure S6: LPR and local energy predictions of models trained on the gallium arsenide (GaAs) dataset, before and after the inclusion of pure Ga structures. (a) shows the ratio in the variance of the committee-predicted local energies ($\Delta^2\tilde{y}_k$) vs. ratio in the LPR, before and after inclusion of the new structures. (b) shows the parity plots of the local energies predicted by a committee of 10 models vs. the committee average prediction, where the points are colored by the corresponding LPR values. Energy values are reported with respect to the single ion energies of Ga and As.

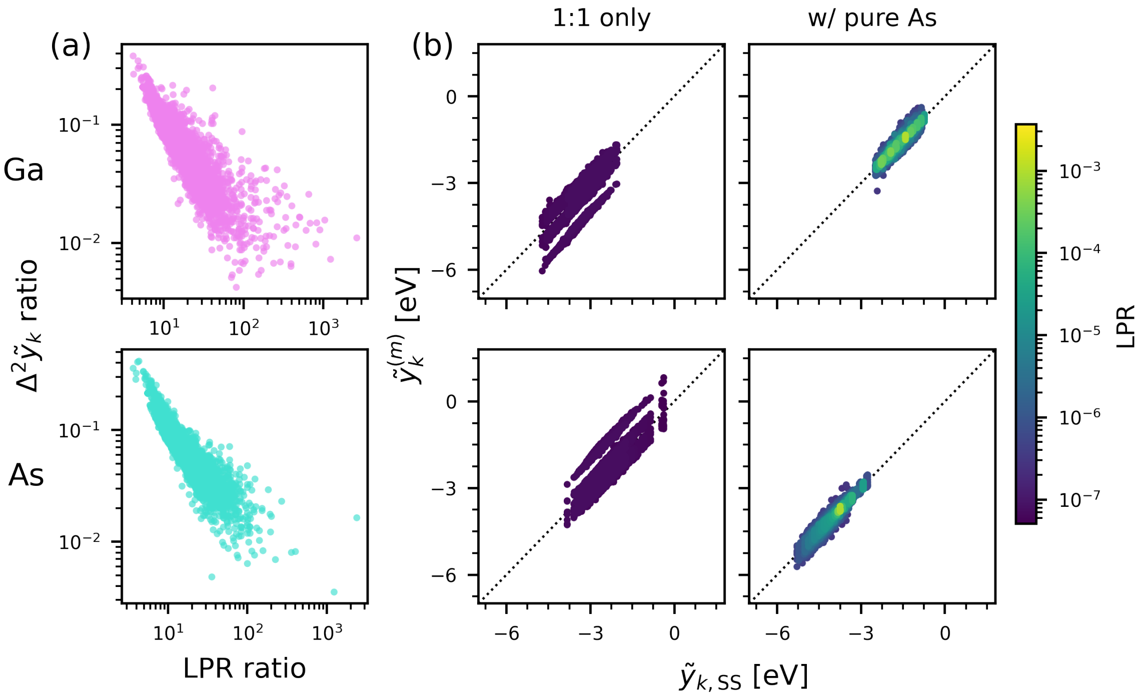

Figure S7: LPR and local energy predictions of models trained on the gallium arsenide (GaAs) dataset, before and after the inclusion of pure As structures. (a) shows the ratio in the variance of the committee-predicted local energies ($\Delta^2\tilde{y}_k$) vs. ratio in the LPR, before and after inclusion of the new structures. (b) shows the parity plots of the local energies predicted by a committee of 10 models vs. the committee average prediction, where the points are colored by the corresponding LPR values. Energy values are reported with respect to the single ion energies of Ga and As.

4 Linear Ridge Regression Model Results for "Case Studies on Real Chemical Systems"

To study whether or not the LPR-related effects demonstrated with sparse kernel models persist for other ML models, the analyses were repeated for the linear ridge regression model. All of the training details are kept identical as the sparse kernel model, except that kernels with respect to a set of reference environments is no longer considered, and the model weights are directly trained with respect to the elements in the SOAP feature vectors. Test set RMSE of the linear ridge regression models are shared below in Table S2. Results, which show that similar effects do indeed persist for the linear ridge regression model, are presented in the below figures.

**Dataset**	case study	RMSE [eV]
**a-Si**	without defects	0.004
	with defects	0.004
**a-C**	1:1 only	0.020
	with diff. ratio	0.018
	with diamond	0.020
**GaAs**	1:1 only	0.008
	with diff. ratio	0.006
	with Pure Ga	0.006
	with Pure As	0.007

Table S2: RMSE of the linear ridge regression models trained on the same datasets as the sparse kernel models from Section 2.

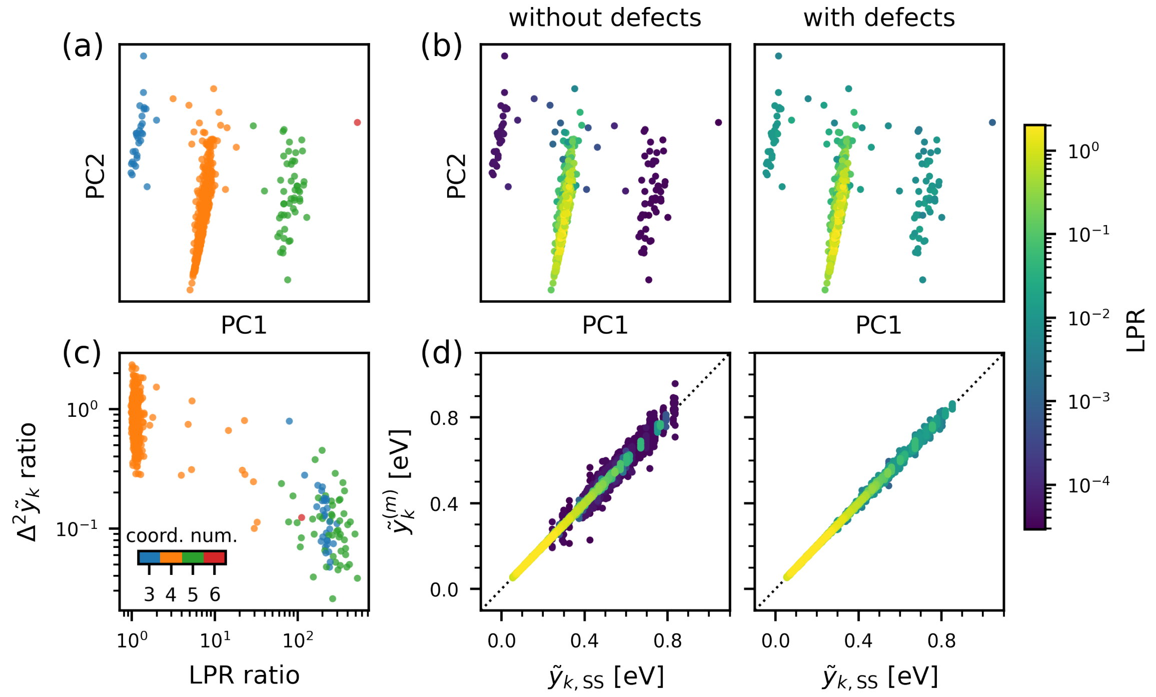

Figure S8: LPR and local energy predictions of linear ridge regression (LRR) models trained on the amorphous silicon (a-Si) dataset before and after the inclusion of structures containing under/over-coordinated defect environments in the training set. (a) shows the principal component analysis (PCA) map with the points colored by the coordination numbers of the atomic environments. (b) shows the PCA map colored by the LPR value from each model. (c) shows the ratio in the variance of the committee-predicted local energies ($\Delta^2\tilde{y}_k$) vs. ratio in the LPR, before and after inclusion of the defect-containing structures in the training set. (d) shows the parity plots of the local energies predicted by a committee of 10 models vs. the committee average prediction, where the points are colored by the corresponding LPR values. Energy values are reported with respect to the atomic energy of crystalline silicon.

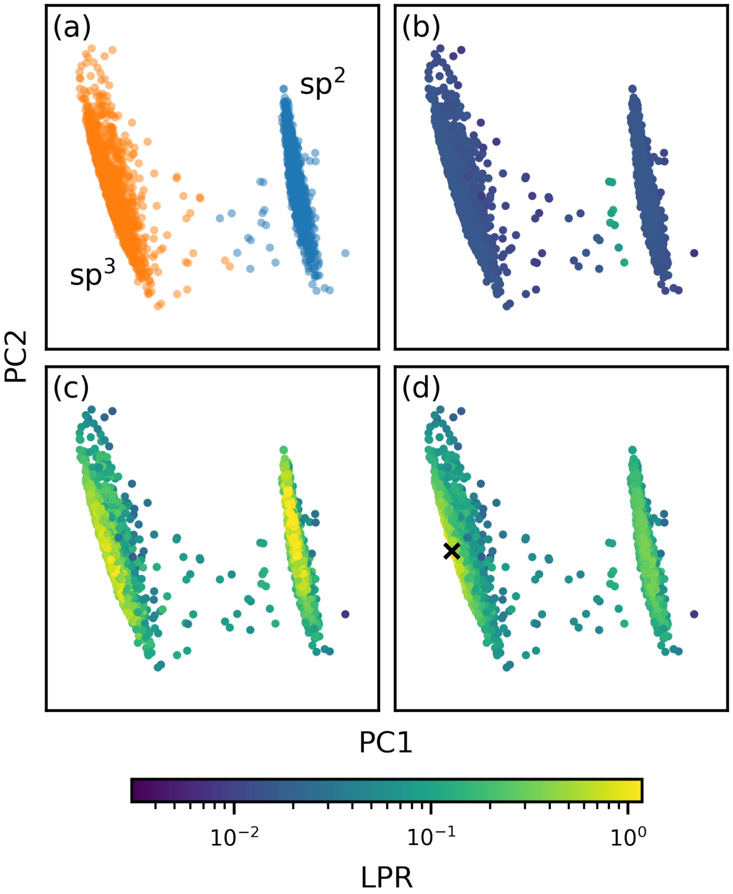

Figure S9: PCA maps for an ensemble of amorphous carbon structures colored by the hybridization of the atoms, shown in (a), and then by the LPR of the linear ridge regression models trained on differently composed training sets. (b) shows the results obtained when the model is trained on a dataset exclusively composed of structures that retain 1:1 ratio between sp^2 and sp^3 carbons. (c) shows the case where 10% of the dataset is replaced with structures exhibiting a different sp^2 to sp^3 ratio. (d) shows the case when a single structure in the dataset is replaced with the crystalline diamond structure, for which the position is marked with a cross.

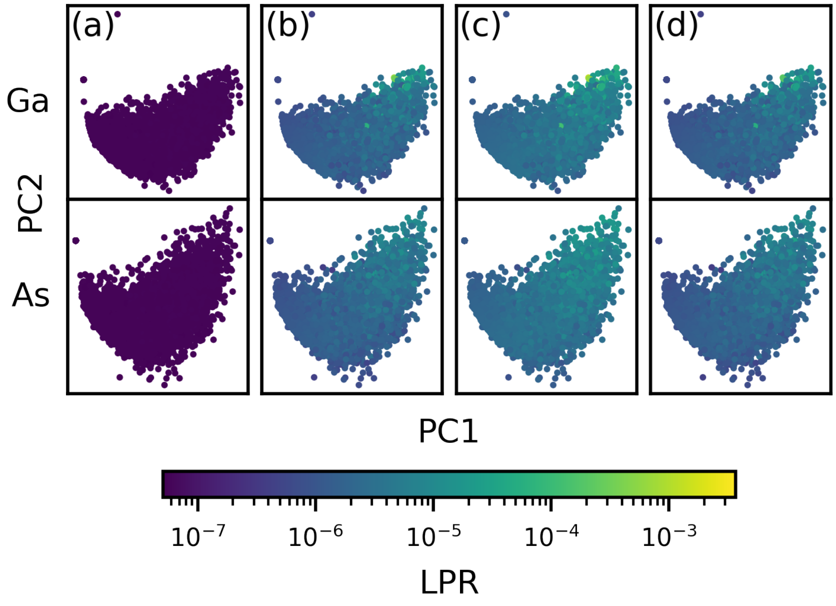

Figure S10: PCA maps for a GaAs dataset. Separate maps are shown for Ga (top row) and As (bottom row) atomic environments, and the points are color-coded by the corresponding LPR values. Results are shared for a series of linear ridge regression models trained on datasets with different compositions: (a) exclusively composed of structures with a Ga:As ratio of 1:1; (b) with 10% of the dataset replaced with structures exhibiting a different Ga:As ratio; (c–d) with 10% of the dataset replaced with pure Ga or pure As structures, respectively.

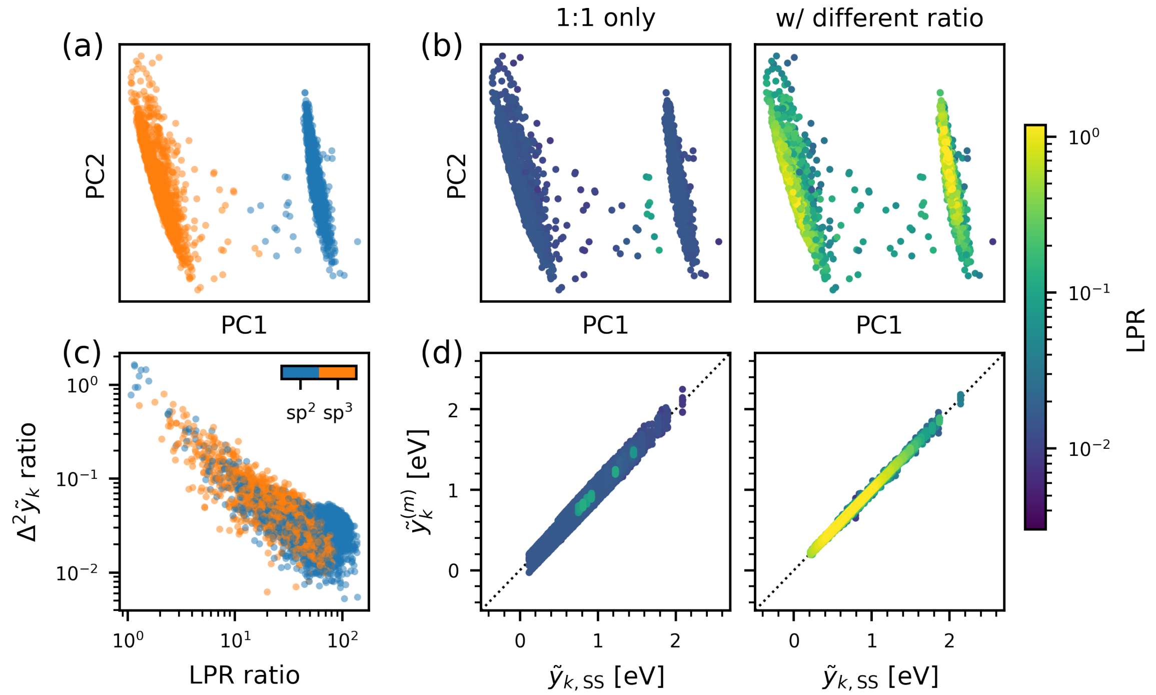

Figure S11: LPR and local energy predictions of linear ridge regression models trained on the amorphous carbon (a-C) dataset before and after the inclusion of structures with a different ratio between sp^2 and sp^3 environments. (a) shows the principal component analysis (PCA) map with the points colored by of the atomic environments, where each point corresponds to an atomic environment in the test set of defect-containing a-Si structures. (b) shows the PCA map colored by the LPR value from each model. (c) shows the ratio in the variance of the committee-predicted local energies ($\Delta^2\tilde{y}_k$) vs. ratio in the LPR, before and after inclusion of the new structures. (d) shows the parity plots of the local energies predicted by a committee of 10 models vs. the committee average prediction, where the points are colored by the corresponding LPR values. Energy values are reported with respect to the atomic energy of diamond.

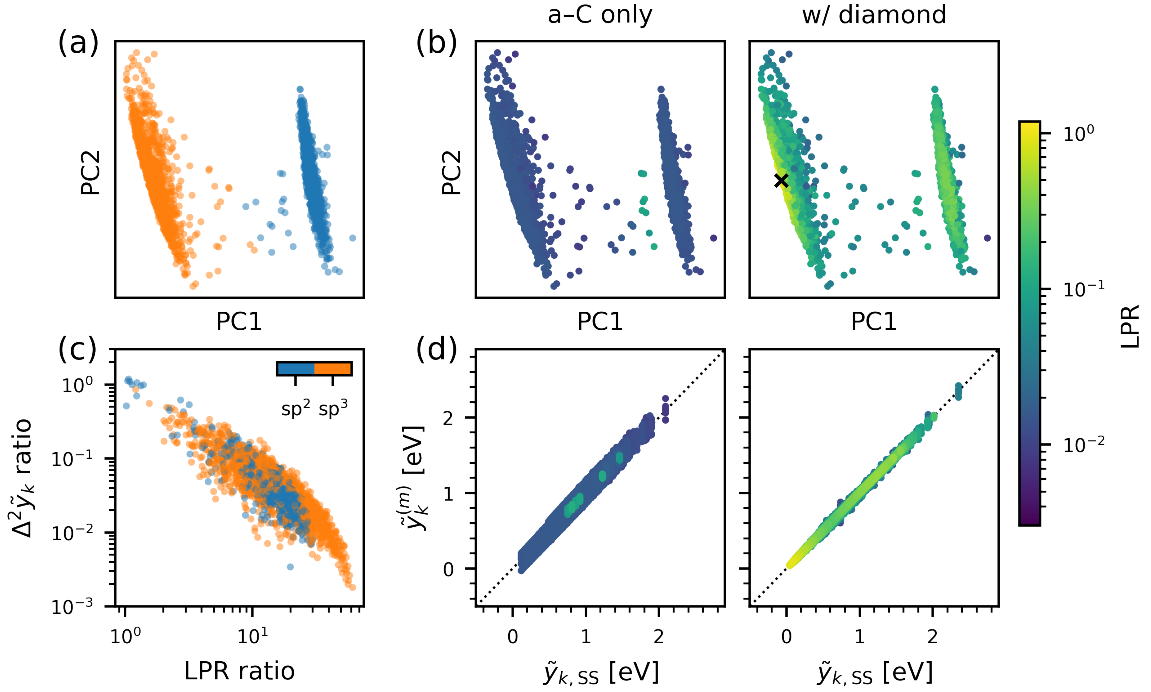

Figure S12: LPR and local energy predictions of models trained on the amorphous carbon (a-C) dataset before and after the inclusion of diamond. (a) shows the principal component analysis (PCA) map with the points colored by of the atomic environments, where each point corresponds to an atomic environment in the test set of defect-containing a-Si structures. (b) shows the PCA map colored by the LPR value from each model. Position of diamond is marked with a cross. (c) shows the ratio in the variance of the committee-predicted local energies ($\Delta^2\tilde{y}_k$) vs. ratio in the LPR, before and after inclusion of diamond. (d) shows the parity plots of the local energies predicted by a committee of 10 models vs. the committee average prediction, where the points are colored by the corresponding LPR values. Energy values are reported with respect to the atomic energy of diamond.

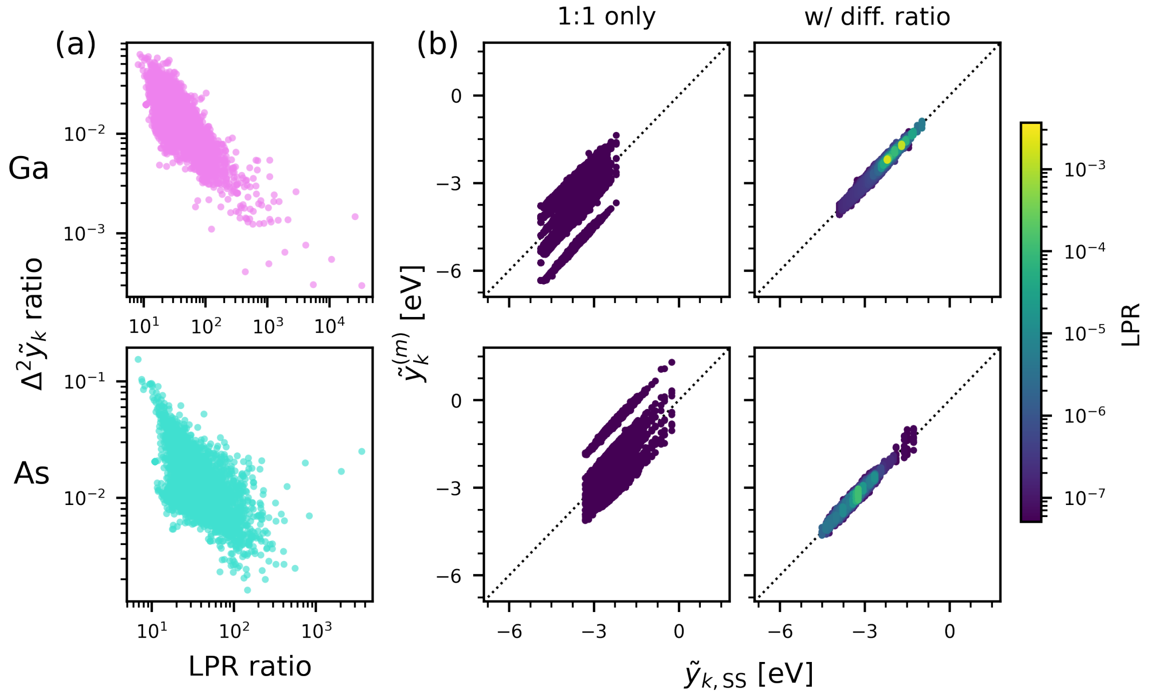

Figure S13: LPR and local energy predictions of linear ridge regression models trained on the gallium arsenide (GaAs) dataset, before and after the inclusion of structures with a different Ga:As ratio. (a) shows the ratio in the variance of the committee-predicted local energies ($\Delta^2\tilde{y}_k$) vs. ratio in the LPR, before and after inclusion of the new structures. (b) shows the parity plots of the local energies predicted by a committee of 10 models vs. the committee average prediction, where the points are colored by the corresponding LPR values. Energy values are reported with respect to the single ion energies of Ga and As.

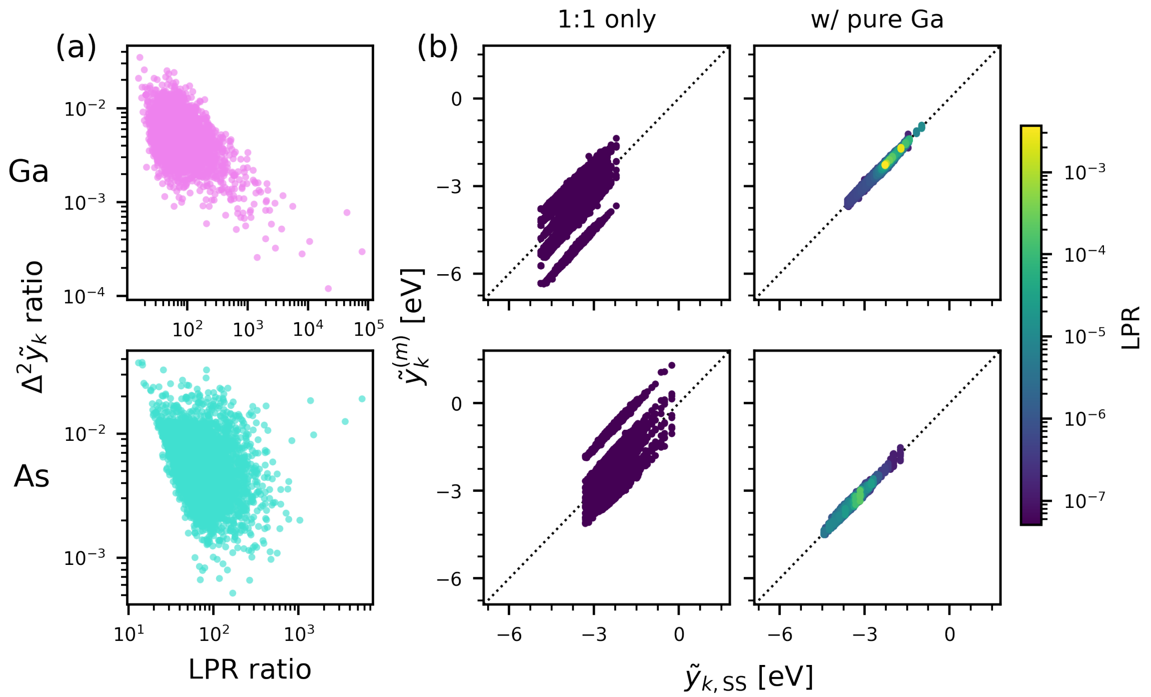

Figure S14: LPR and local energy predictions of linear ridge regression models trained on the gallium arsenide (GaAs) dataset, before and after the inclusion of pure Ga structures. (a) shows the ratio in the variance of the committee-predicted local energies ($\Delta^2\tilde{y}_k$) vs. ratio in the LPR, before and after inclusion of the new structures. (b) shows the parity plots of the local energies predicted by a committee of 10 models vs. the committee average prediction, where the points are colored by the corresponding LPR values. Energy values are reported with respect to the single ion energies of Ga and As.

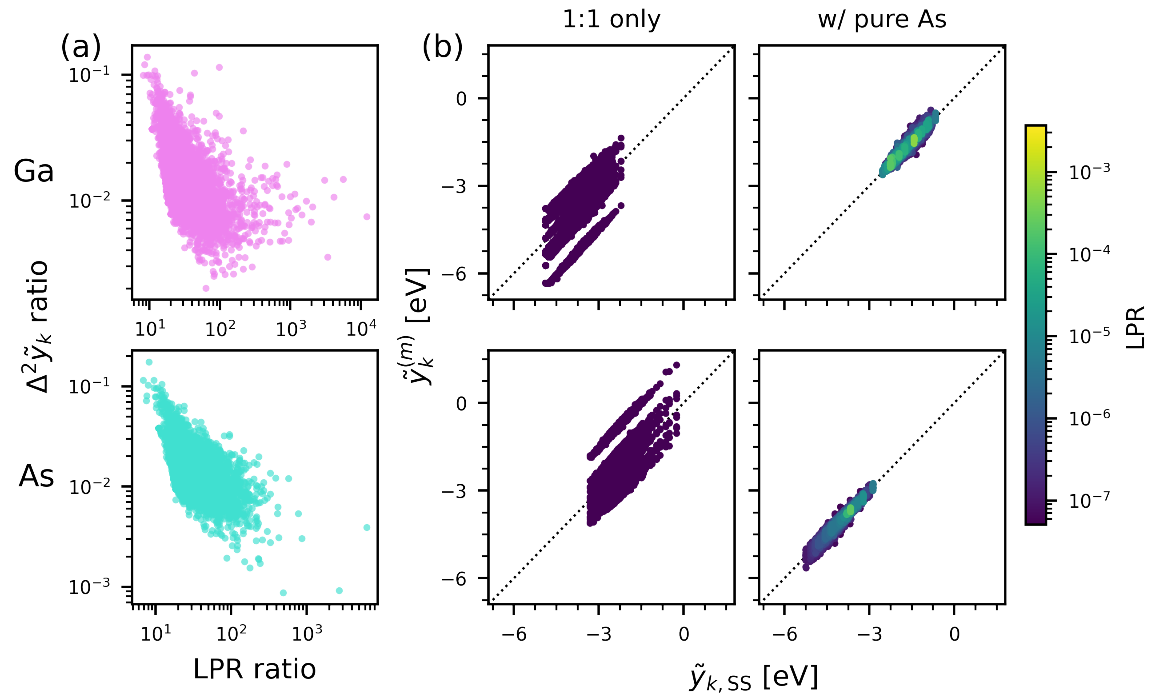

Figure S15: LPR and local energy predictions of linear ridge regression models trained on the gallium arsenide (GaAs) dataset, before and after the inclusion of pure As structures. (a) shows the ratio in the variance of the committee-predicted local energies ($\Delta^2\tilde{y}_k$) vs. ratio in the LPR, before and after inclusion of the new structures. (b) shows the parity plots of the local energies predicted by a committee of 10 models vs. the committee average prediction, where the points are colored by the corresponding LPR values. Energy values are reported with respect to the single ion energies of Ga and As.

5 Details of ML Model Training for "More Realistic Applications"

Here, the ML models were trained to be equivalent to the Gaussian approximation potential (GAP) model developed by Deringer et al.[5] Sparse kernel models[1] were hence trained to predict the total energies per atom of the chemical structures by summing together, then taking the mean of, the local energy contributions of the constituent atoms. As stated before, averaging out of the target and the prediction is done to ensure that the regularization strength is kept consistent between the structures, which may contain different numbers of atoms. The local environments of the atoms were represented using SOAP, computed with rascaline. Identical set of hyperparameters from the previous case study on a-C was used, with the exception of the interaction cutoff that has been re-adjusted to 3.7 Å (see Table S1). Feature vectors were normalized as done in the reference study. 1000 amorphous bulk structures were randomly sampled from the published dataset,[9] and 1000 reference environments were selected from the sampled structures using the FPS method. Models were trained by performing a grid search to find the regularzation strength that minimizes the RMSE on a validation set of 100 separately sampled structures. Four different models were trained: first one on the original dataset of 1000 amorphous bulk structures, second one where 10 structures are replaced with diamond and its variant, single-environment structures, third one where 10 structures are replaced with graphite and its variant, single-environment structures, and final one where 5 structures are replaced with diamond and its variant, single-environment structures, and another 5 with graphite and its variant, single-environment structures. The models achieved test set RMSEs (and % RMSEs) of 0.066 eV (12%), 0.077 eV (13%), 0.066 eV (11%), and 0.078 eV (14%), respectively.

By single-environment structures, we refer to the structures in which, due to their symmetry and homogeneity, all the local environments described with the same, identical feature vector. As detailed in the manuscript, these structures have a well-defined local prediction target, and hence inclusion of such structures lead to a maximal value in the LPR for the corresponding local environment. While there exist several examples of such single-environment structures, such as the crystalline diamond or graphite, it is desirable to be able to generate a multitude of such structures that are similar to the actual local environments of interest. This is attainable by starting with a known single-environment structure, then distorting the unit cell parameters whilst continuing to occupy the single Wyckoff position of the original structure. Two-dimensional depiction of the explained approach is provided in Figure S16. This ensures that while the local environment is varied, all of the local environments in the structures are still equivalent to one another. In practice, to generate the variants of diamond and graphite (original structures obtained from Materials Project under reference codes mp-66 and mp-48, respectively) for this case study, random offsets of $\pm 10°$ were sampled from a uniform distribution and applied to α, β, and γ of the original unit cell. Then, ± 0.25 Å were sampled from a uniform distribution and applied to a and b. The final parameter c was then calculated to be the value that conserves the original unit cell volume. We note that the choice to conserve the unit cell volume is not a requirement to retain the single-environment nature of the resulting structures. However, it was done to ensure relative similarity of the resulting variant structures to the original diamond or graphite structures.

To provide a more practical example in which an increase in the LPR leads to improved model transferability, a modified case study was conducted with a-C. Here, instead of training on the entire density range of the a-C structures available, the initial dataset was composed of 1000 high density structures with the density between 2.9 and 3.6 g/cm^3. Then, two revised datasets were constructed, where 1% of the dataset was replaced with either graphite-like high symmetry structures or low density (< 2.1 g/cm^3) a-C structures, respectively. Three models were trained on these different datasets, adopting the same parameters and model training details as described above. Note that the RMSE on a test set of 100 high density, bulk a-C structures was 0.056 eV, 0.055 eV, and 0.056 eV for the three models (%RMSE of 9% in all cases). For the three models, their transferability to the out-of-sample surface-containing structures of Deringer et al.[10] was considered. In doing so, we first calculated the LPR for the surface structures, an example of which is shown in Figure S17. In the original model trained only on the high density a-C structures, LPR is considerably low for the atoms at the surface. In the other two models, while the surface atoms still exhibit lower LPR than the atoms at the "core", the LPR for these surface atoms is notably higher than that of the original model. Figure S18 shows the total energy per atom predictions of the three models for the surface-containing structures. It

is clear that better transferability is observed for the two models where the dataset was modified to include the graphite-like structures or the low density a-C structures. Indeed, the RMSE for the 50 surface-containing structures decreases from 722 meV of the original model to 446 meV and 167 meV for the two models, respectively. This thus provides an example of the association between higher robustness of the local predictions and the resulting model transferability.

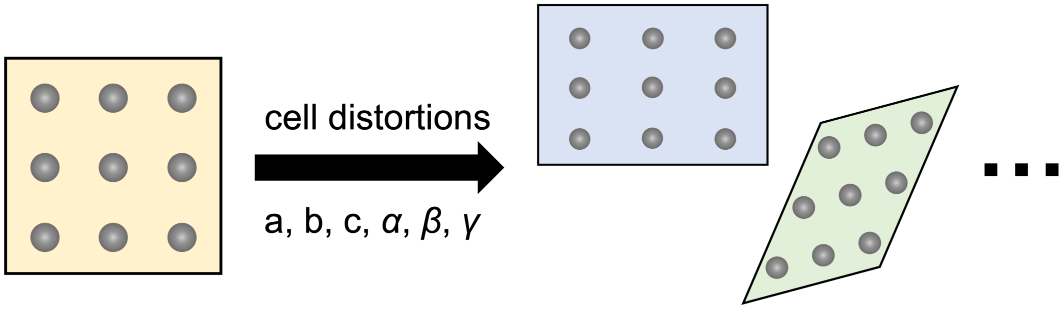

Figure S16: Two-dimensional depiction of how the variant single-environment structures can be generated starting from a known one. The original single-environment structure to the left is comprised of nine atoms positioned in a perfectly equidistant lattice. The unit cell parameters of the original structure can be distorted whilst occupying the original Wyckoff position. In the resulting variant structures shown on the right, atoms of the unit cell still exhibit equivalent local environments amongst themselves.

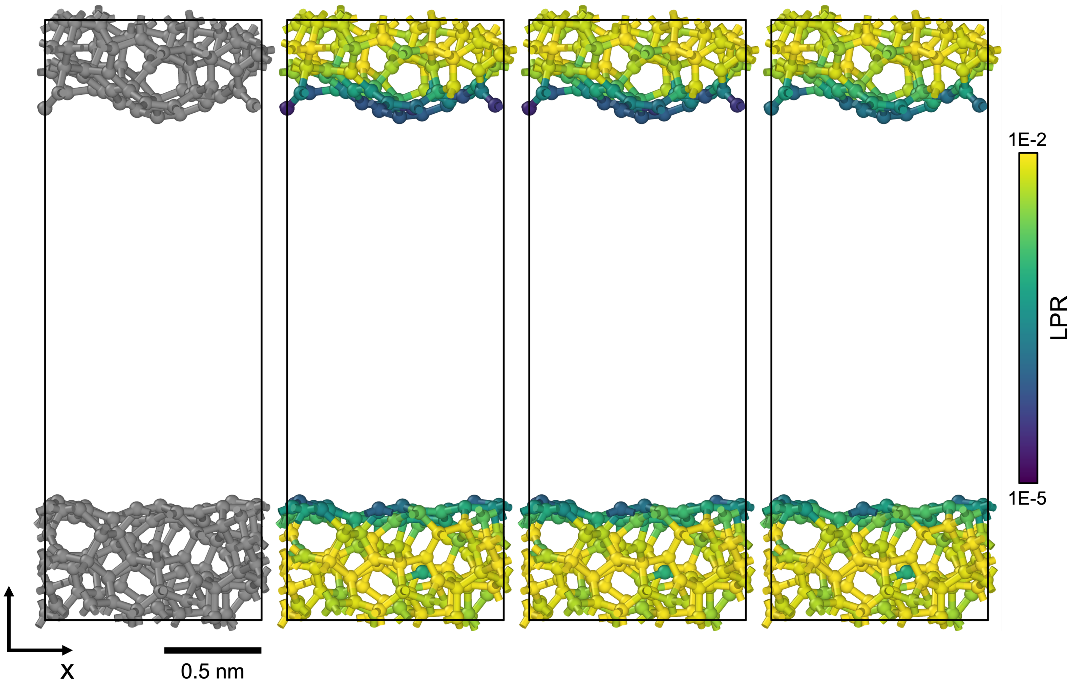

Figure S17: Configuration of the surface-containing amorphous carbon structure used in the modified case study described above. Far left panel shows the example surface structure. The second panel shows the structure colored by the local prediction rigidity (LPR) of a model trained on 1000 high density bulk a-C strucures. The third and fourth panels show the LPRs of models where the 1% of the original training set was replaced with either graphite-like high symmetry structures (third) or low density bulk a-C structures (fourth). Increase in the LPR with modifications to the training set can be observed, especially at the top surface.

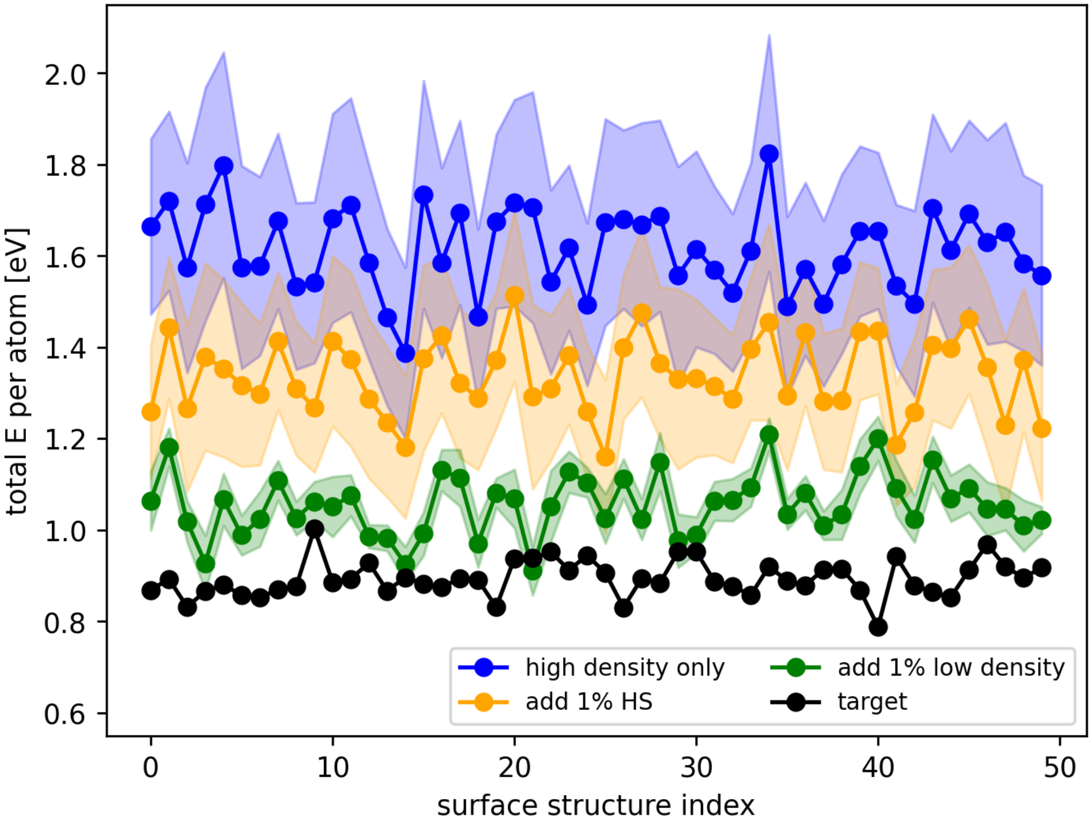

Figure S18: Total energy per atom predictions of the models appearing in the modified case study. For the three models, standard deviation across a committee of 10 models trained by subsampling of the corresponding dataset is also shown. Ground truth values from the DFT calculations on the surface-containing 216-atom structures are also presented. Reported energy values are zeroed at the energy per atom of crystalline diamond.

6 Details of ML Model Training for "Extension to Neural Network Models"

For the neural network (NN) model training, the exact same dataset as the one used to train the sparse kernel model in Section 3 was taken. The NN model was constructed and trained using PyTorch[11] with double precision. The Behler–Parinello approach of having the NN model predict on the locally decomposed energies then summing together the contributions to regress on the global target quantity was adopted. SOAP feature vectors were fed into the input layer, and the corresponding local energy was predicted at the output layer. Between the input and output layers, two hidden layers with 16 nodes in each layer were placed. At each node, incoming values were put through a sigmoid activation function. The layers were then fully connected sequentially, resulting in a NN model with 9488 weight parameters. To ensure applicability of the LPR formulation, explicit L^2 regularization was performed, rather than imposing an early stopping criterion with respect to a validation set. Adam optimizer[12] was used with a learning rate of 0.005, which was modified during the training process using the "ReduceLROnPlateau" scheduler implemented internally in PyTorch with the following parameters: factor = 0.5, patience = 100, cooldown = 100, eps = 10^{-8}. Model training was carried out for 25,000 epochs to ensure that the model, though trained using a numerical stochastic gradient method, is as close to the true minimum of the loss function as possible. As the regularization strength directly impacts the resulting LPR, same regularization strength as the sparse kernel model was used in model training to ensure comparability between the two models. The NN model achieved a test set RMSE of 0.070 eV (%RMSE = 12%), which is comparable to that of the sparse kernel model. For analysis, two additional NN models were trained: one employing the early stopping criterion with respect to a validation set in model training, and another where the nonlinear activation functions are removed to retain linearity in the entire network.

7 Supplementary Figures for "Extension to Neural Network Models"

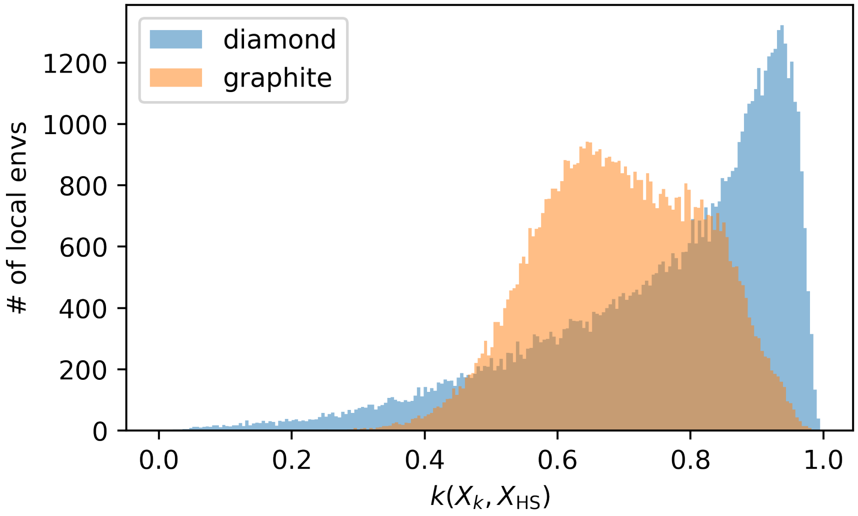

Figure S19: Histograms of the SOAP kernel similarity between the local environment and diamond or graphite, for the local environments that appear in the training set of 1000 amorphous bulk structures used in this study.

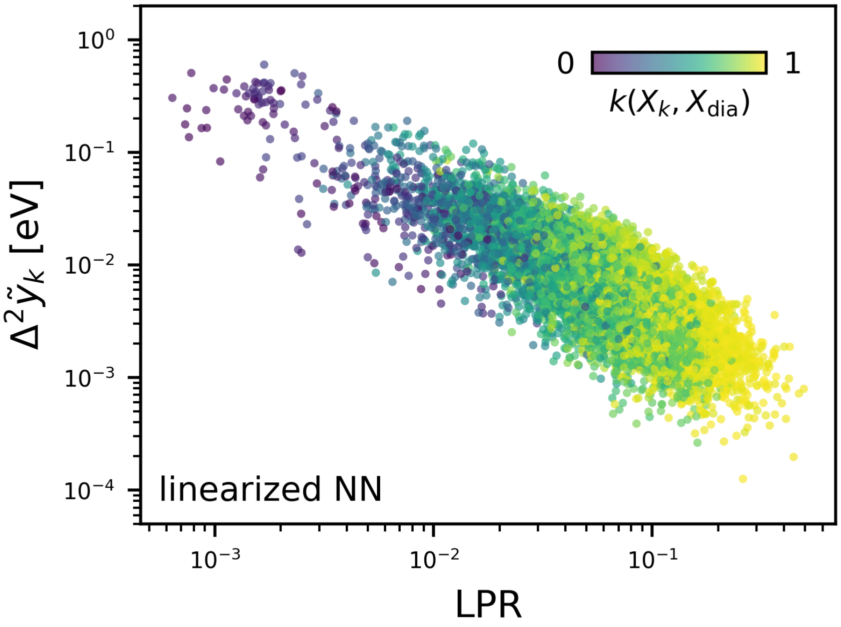

Figure S20: $\Delta^2\tilde{y}_k$ for a committee of 10 models vs. the LPR, calculated for the low density carbon film using the NN model with linear activation function, or no sigmoid activation at the nodes.

References

(1) Deringer, V. L.; Bartók, A. P.; Bernstein, N.; Wilkins, D. M.; Ceriotti, M.; Csányi, G. Gaussian Process Regression for Materials and Molecules. *Chem. Rev.* **2021**, *121*, 10073–10141.

(2) Willatt, M. J.; Musil, F.; Ceriotti, M. Feature Optimization for Atomistic Machine Learning Yields a Data-Driven Construction of the Periodic Table of the Elements. *Phys. Chem. Chem. Phys.* **2018**, *20*, 29661–29668.

(3) Bartók, A. P.; Kermode, J.; Bernstein, N.; Csányi, G. Machine Learning a General-Purpose Interatomic Potential for Silicon. *Phys. Rev. X* **2018**, *8*, 041048.

(4) Deringer, V. L.; Bernstein, N.; Bartók, A. P.; Cliffe, M. J.; Kerber, R. N.; Marbella, L. E.; Grey, C. P.; Elliott, S. R.; Csányi, G. Realistic Atomistic Structure of Amorphous Silicon from Machine-Learning-Driven Molecular Dynamics. *J. Phys. Chem. Lett.* **2018**, *9*, 2879–2885.

(5) Deringer, V. L.; Csányi, G. Machine Learning Based Interatomic Potential for Amorphous Carbon. *Phys. Rev. B* **2017**, *95*, 094203.

(6) Imbalzano, G.; Ceriotti, M. Modeling the Ga/As Binary System across Temperatures and Compositions from First Principles. *Phys. Rev. Materials* **2021**, *5*, 063804.

(7) Imbalzano, G.; Ceriotti, M. Modeling the Ga/As binary system across temperatures and compositions from first principles, 2021.

(8) Lopanitsyna, N.; Fraux, G.; Springer, M. A.; De, S.; Ceriotti, M. Modeling high-entropy transition metal alloys with alchemical compression. *Phys. Rev. Mater.* **2023**, *7*, 045802.

(9) Deringer, V. L.; Csanyi, G. Research data supporting "Machine learning based interatomic potential for amorphous carbon", 2017.

(10) Deringer, V. L.; Caro, M. A.; Jana, R.; Aarva, A.; Elliott, S. R.; Laurila, T.; Csányi, G.; Pastewka, L. Computational Surface Chemistry of Tetrahedral Amorphous Carbon by Combining Machine Learning and Density Functional Theory. *Chem. Mater.* **2018**, *30*, 7438–7445.

(11) Paszke, A.; Gross, S.; Massa, F.; Lerer, A.; Bradbury, J.; Chanan, G.; Killeen, T.; Lin, Z.; Gimelshein, N.; Antiga, L.; Desmaison, A.; Kopf, A.; Yang, E.; DeVito, Z.; Raison, M.; Tejani, A.; Chilamkurthy, S.; Steiner, B.; Fang, L.; Bai, J.; Chintala, S. PyTorch: An Imperative Style, High-Performance Deep Learning Library. *Adv. Neural Inf. Process.* **2019**, *32*, 8024–8035.

(12) Kingma, D. P.; Ba, J. Adam: A Method for Stochastic Optimization. *arXiv preprint arXiv:1412.6980* **2023**.
